# Regulation of ferroptosis by PI3K/Akt signaling pathway: a promising therapeutic axis in cancer

**DOI:** 10.3389/fcell.2024.1372330

**Published:** 2024-03-18

**Authors:** Hua Su, Chao Peng, Yang Liu

**Affiliations:** ^1^ Xingyi People’s Hospital, Xinyi, China; ^2^ The Second Affiliated Hospital of Zhengzhou University, Zhengzhou, China

**Keywords:** PI3K/AKT, ferroptosis, cancer, cell death, PI3K, Akt

## Abstract

The global challenge posed by cancer, marked by rising incidence and mortality rates, underscores the urgency for innovative therapeutic approaches. The PI3K/Akt signaling pathway, frequently amplified in various cancers, is central in regulating essential cellular processes. Its dysregulation, often stemming from genetic mutations, significantly contributes to cancer initiation, progression, and resistance to therapy. Concurrently, ferroptosis, a recently discovered form of regulated cell death characterized by iron-dependent processes and lipid reactive oxygen species buildup, holds implications for diseases, including cancer. Exploring the interplay between the dysregulated PI3K/Akt pathway and ferroptosis unveils potential insights into the molecular mechanisms driving or inhibiting ferroptotic processes in cancer cells. Evidence suggests that inhibiting the PI3K/Akt pathway may sensitize cancer cells to ferroptosis induction, offering a promising strategy to overcome drug resistance. This review aims to provide a comprehensive exploration of this interplay, shedding light on the potential for disrupting the PI3K/Akt pathway to enhance ferroptosis as an alternative route for inducing cell death and improving cancer treatment outcomes.

## 1 Introduction

Globally, cancer presents a substantial health challenge characterized by elevated rates of occurrence and death, with approximately 19.3 million new cases and 10 million fatalities recorded in 2020. The projected surge in worldwide cancer instances to 28.4 million by 2040, especially in countries undergoing transition, emphasizes the escalating burden of the disease (Sung et al., 2021). The PI3K/Akt signaling pathway, a crucial intracellular cascade involving phosphatidylinositol-3-kinase (PI3K) and Akt (protein kinase B), is frequently amplified in various human malignancies. This pathway, activated by external stimuli like growth factors and hormones, regulates essential cellular processes such as cell proliferation, programmed cell death (apoptosis), and angiogenesis. Alterations in this pathway, often stemming from gene mutations in *PIK3CA* and loss of *PTEN*, significantly contribute to cancer initiation, progression, and resistance to therapeutic agents. The dysregulation manifests as aberrant phosphorylation events and disruptions mediated by microRNAs. Notably, the influence of the PI3K/Akt pathway on multidrug resistance emphasizes its central role in cancer therapy (Yang et al., 2019; Rascio et al., 2021; Peng et al., 2022). Ferroptosis is a newly discovered form of regulated cell death (RCD) characterized by processes dependent on iron, resulting in the accumulation of lipid reactive oxygen species (ROS). Morphological distinctions of ferroptosis from apoptosis and necrosis include diminished mitochondrial volume, heightened membrane density, modified mitochondrial cristae, and, notably, the “ballooning phenotype” observed in some cancer cells, such as ovarian cells (Battaglia et al., 2022). The ballooning phenotype in ferroptosis refers to the swelling or ballooning of cellular organelles, particularly mitochondria and endoplasmic reticulum, caused by lipid peroxidation and membrane damage. This morphological change, characterized by forming a clear, rounded cell consisting mainly of empty cytosol, is specific to cells undergoing ferroptosis (Battaglia et al., 2020; Battaglia et al., 2022; Battaglia et al., 2023). Key features encompass glutathione depletion, the suppression of glutathione peroxidase 4 (GPX4), leading to lipid peroxidation, and the generation of reactive oxygen species facilitated by iron. Ferroptosis can be triggered by various substances and is implicated in diseases such as cancer (Li et al., 2020; Chen J. et al., 2023). Due to the novelty of this type of cell death, the mechanisms involved in its regulation are being studied. The relationship between signaling pathways and ferroptosis constitutes a dynamic regulatory system with immense implications for therapeutic approaches and disease management. Recently, the interplay between inflammatory pathways, such as JAK-STAT, NF-κB, cGAS-STING, and MAPK, with ferroptosis has been highlighted. This interplay influences cellular behavior and impacts critical processes such as iron homeostasis (Chen Y. et al., 2023). Given that the PI3K/Akt pathway is dysregulated across various cancer types, establishing its correlation with ferroptosis can provide valuable insights into the detailed mechanisms contributing to this specific mode of cell death (Yang et al., 2019). Investigating the crosstalk between PI3K/Akt dysregulation and ferroptosis may uncover the intricate molecular interactions and signaling cascades that drive or inhibit ferroptotic processes in cancer cells. Evidence indicates that inhibition of this signaling pathway could sensitize cancer cells to ferroptosis induction (Yi et al., 2020). Targeting the PI3K/Akt pathway, which is prevalently overactivated in various cancers, holds promise for overcoming drug resistance. The PI3K/Akt pathway, linked to cell survival and resistance mechanisms, can be disrupted to sensitize cancer cells to ferroptosis, offering an alternative route to induce cell death that may bypass traditional resistance mechanisms. This strategy, if successful, could represent an innovative therapeutic approach to enhance cancer treatment outcomes, particularly in cases of drug-resistant cancers. This review aims to uncover the interaction between this signaling pathway and ferroptosis in carcinogenesis for the first time, providing insights into potential therapeutic strategies targeting both the PI3K/Akt pathway and ferroptosis.

## 2 A quick overview of ferroptosis

**FIGURE 1:**
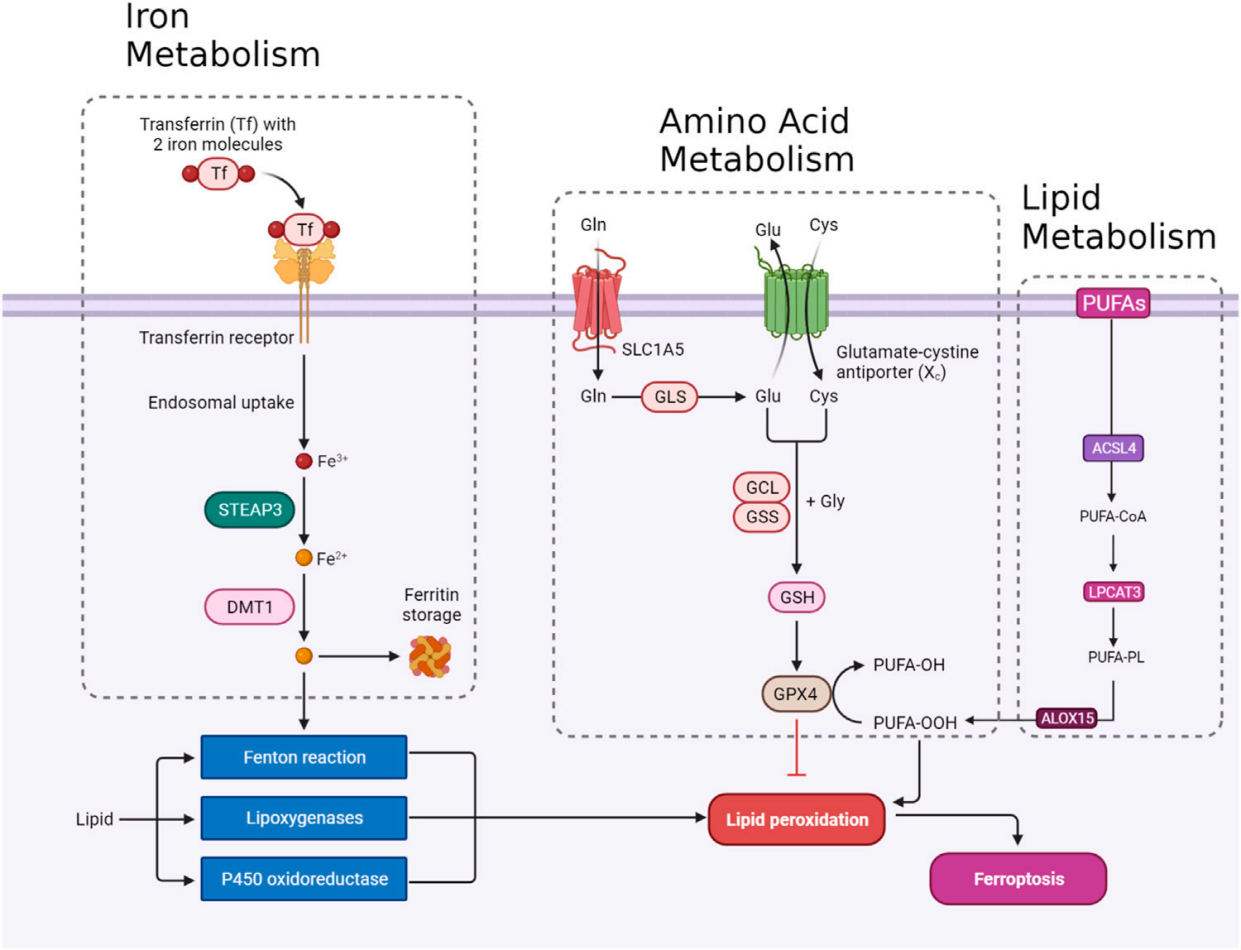
An overview of ferroptosis pathways including amino acid, lipid, and iron metabolism.

Ferroptosis, a tightly regulated form of programmed cell death, is orchestrated by a complex interaction of molecular mechanisms across three primary pathways: the amino acid metabolism (GPX4-modulated) pathway, iron metabolism pathway, and lipid metabolism pathway (Figure 1). The amino acid pathway involves the inhibition of GPX4, a crucial enzyme in the antioxidant defense system, either directly or indirectly by glutathione depletion. This inhibition leads to the accumulation of phospholipid hydroperoxides, which inflict severe damage to the cell membrane and ultimately trigger ferroptosis. The iron metabolism pathway depends on the labile iron pool (LIP), a reservoir of redox-active iron. This LIP facilitates ROS production through Fenton reactions, contributing to the initiation of lipid peroxidation, a hallmark of ferroptosis. The lipid metabolism pathway emphasizes the significant role of lipid peroxidation in ferroptosis, encompassing both non-enzymatic and enzymatic processes. Enzymes such as acyl-CoA synthetase long-chain family member 4 (ACSL4), lipoxygenases (LOX), and NADPH oxidase reductase hold integral positions. Cancer cells exhibit susceptibility to ferroptosis due to diverse factors, including alterations in metabolism, genetic mutations, and imbalances in ferroptosis defense mechanisms. Metabolic shifts, like increased polyunsaturated fatty acid-phospholipids (PUFA-PLs) during epithelial-to-mesenchymal transition (EMT), render certain cancer cells sensitive to ferroptosis. Various cancer types, such as clear-cell renal cell carcinoma (ccRCC) (Zou et al., 2020), non-neuroendocrine small-cell lung cancer (SCLC) (Bebber et al., 2021), and triple-negative breast cancer (TNBC) (Verma et al., 2020), inherently possess susceptibility to ferroptosis based on their distinctive metabolic characteristics. Surprisingly, certain genetic mutations, usually associated with ferroptosis resistance, can paradoxically make cancer cells vulnerable to ferroptosis. Examples include inactivation of the E-cadherin–neurofibromin 2 (NF2)-Hippo signaling axis and loss of the Von Hippel-Lindau (VHL) tumor suppressor. Oncogene activation, such as epidermal growth factor receptor (EGFR) or isocitrate dehydrogenase 1 (IDH1) mutations, may either suppress or promote ferroptosis based on specific circumstances. An imbalance in ferroptosis defense systems involving GPX4-dependent and GPX4-independent arms creates vulnerabilities in cancer cells. For instance, low expression of ferroptosis defense genes like FSP1, DHODH, or GPX4 can make cancer cells highly dependent on the remaining defense arm, providing potential targets for inducing ferroptosis in specific cancer types (Lei et al., 2022). Understanding these complex pathways provides invaluable insights for developing therapeutic strategies, particularly in diseases like cancer, where ferroptosis plays a prominent role (Figure 1). Modulating key components of these pathways holds immense promise for controlling ferroptosis and addressing diverse pathological conditions (Zhang C. et al., 2022).

### 2.1 Amino acid metabolism pathway

#### 2.1.1 System Xc^-^

The integrity of redox balance relies significantly on System Xc^−^, which comprises the SLC7A11 (xCT) cystine/glutamate antiporter and the regulatory subunit SLC3A2 (4F2hc). This system plays a critical role by absorbing cystine and subsequently converting it into cysteine for the biosynthesis of glutathione (GSH), a fundamental antioxidant. GSH serves as a vital cofactor for GPX4, the principal downstream element that hinders lipid peroxidation. Disrupting intracellular cysteine and GSH levels during ferroptosis negatively impacts GPX4 activity, resulting in cellular demise. The regulation of System Xc^−^ encompasses transcription factors like ATF4 and Nrf2, epigenetic modifications, post-translational mechanisms, and signaling pathways. Small molecular disruptors like Erastin can influence System Xc^−^, prompting ferroptosis. A profound understanding of these regulatory processes is imperative for formulating effective treatments to target this axis, particularly in combating drug-resistant solid tumors (Figure 1) (Li F-J. et al., 2022).

#### 2.1.2 GPX4

GPX4 occupies a pivotal position in the machinery that governs ferroptosis. GPX4, a selenoprotein with exceptional antioxidant properties, stands as the master regulator of ferroptosis. The specific molecular function of GPX4 in ferroptosis involves its ability to reduce complex hydroperoxides, including phospholipid hydroperoxides and cholesterol hydroperoxides, to their non-toxic counterparts. The reduction of lipid hydroperoxides interrupts the chain reaction of lipid peroxidation, which is a crucial feature of ferroptosis. GPX4 achieves this function by utilizing glutathione (GSH) as a cofactor. In addition to reducing lipid hydroperoxides, GPX4 uniquely converts polyunsaturated fatty acid hydroperoxides (PUFA-OOH) to their corresponding alcohols (PUFA-OH). This function is crucial for interrupting the chain reaction of lipid peroxidation. GSH is an antioxidant essential for the normal physiological function of GPX4. Disruptions in GSH synthesis, cysteine availability, or GPX4 function can either sensitize cells to ferroptosis or directly trigger this lethal form of cell death (Figure 1) (Seibt et al., 2019).

#### 2.1.3 NRF2

Nuclear factor erythroid 2-related factor 2 (NRF2) is a crucial transcription factor in safeguarding cells against oxidative stress. In typical circumstances, NRF2 is confined to the cytoplasm by its inhibitor, Keap1. However, when exposed to oxidative stress or electrophilic challenges, Keap1 undergoes modifications, causing the release and movement of NRF2 into the nucleus. Once within the nucleus, NRF2 attaches to antioxidant response elements (AREs) in the promoter regions of its target genes, initiating the transcription of diverse antioxidant and cytoprotective proteins. One such target gene regulated by NRF2 is *GPX4*. As a downstream effector of NRF2, GPX4 plays a role in cellular defense against lipid peroxidation, a process where reactive species target polyunsaturated fatty acids, generating harmful lipid peroxides. Consequently, the NRF2-GPX4 axis serves as a crucial mechanism in preserving cellular redox homeostasis and defending against oxidative damage, as well as ferroptosis (Figure 1) (Dodson et al., 2019).

### 2.2 Iron metabolism

#### 2.2.1 Transferrin/transferrin receptor

Transferrin (TF) and its receptor (TFRC/TFR1) play vital roles in iron metabolism and are implicated in ferroptosis. TF, a glycoprotein, conveys iron from the bloodstream to tissues by binding reversibly to iron, releasing iron in endosomes in a pH-dependent manner. TF is crucial in triggering ferroptosis during amino acid deprivation, and its deficiency is associated with conditions like atransferrinemia. TFRC, a receptor for iron-loaded TF, facilitates the internalization of the TF-TFRC complex, leading to subsequent iron release in endosomes, contributing to cellular iron equilibrium (Figure 1). The expression of TFRC in cancer cells is correlated with heightened iron requirements for cell proliferation, positioning it as a potential target for inducing ferroptosis in cancer treatment (Chen et al., 2020; Feng et al., 2020).

#### 2.2.2 STEAP3/DMT1

The detailed processes of the STEAP3/DMT1 axis are central to the homeostasis of iron ions in the cell, orchestrating a sequence of events to maintain proper iron balance and distribution. Iron, a vital trace element, undergoes dynamic transformations to fulfill physiological needs. The journey begins with the generation of Fe^2+^, which can arise from intestinal absorption or the breakdown of erythrocytes. Subsequently, ceruloplasmin catalyzes the oxidation of Fe^2+^ to Fe^3+^, leading to the formation of TF-Fe^3+^ on the cell membrane. This complex engages with the membrane protein TF receptor 1 (TFR1), initiating its endocytosis. Within the cellular environment, the six-transmembrane epithelial antigen of the prostate 3 (STEAP3) assumes a crucial role by reducing Fe^3+^ back to Fe^2+^. This reduction is an integral step in the regulation of iron ions, allowing the transformed Fe^2+^ to be strategically stored. The labile iron pool (LIP) and ferritin serve as reservoirs for the stored Fe^2+^. The transport of Fe^2+^ to these storage compartments is facilitated by the divalent metal transporter 1 (DMT1) (Figure 1). This transporter ensures the efficient incorporation of Fe^2+^ into both the labile iron pool and ferritin, contributing to the complex system of iron regulation (Bogdan et al., 2016; Yanatori and Kishi, 2019; Li et al., 2020).

#### 2.2.3 Ferritin

Ferritin, an essential cytosolic protein responsible for storing iron, is a complex composed of two integral subunits: ferritin heavy chain 1 (FTH1) and ferritin light chain (FTL). FTH1 plays a fundamental role within this dynamic system due to its possession of ferroxidase activity. This enzymatic function is critical for converting ferrous iron (Fe^2+^) to ferric iron (Fe^3+^). This conversion is instrumental in incorporating iron into the ferritin mineral core. FTL collaborates with FTH1 in this process, working synergistically to facilitate the orderly entry of iron ions into the ferritin structure. The concerted action of these subunits ensures efficient iron storage within the ferritin complex, contributing to the overall regulation and balance of intracellular iron levels. This sophisticated mechanism not only safeguards against iron-induced toxicity but also enables the controlled release of iron when needed, reflecting the crucial role of ferritin in cellular iron homeostasis (Figure 1) (Tian et al., 2020a; Chen et al., 2020; Liu J. et al., 2022).

#### 2.2.4 NCOA4

Selective autophagy mediated by nuclear receptor coactivator 4 (NCOA4) is the predominant process for releasing iron from ferritin. This mechanism involves NCOA4 binding to ferritin and directing it to lysosomes for degradation, facilitating iron release. Cellular iron levels regulate NCOA4 levels, consequently affecting the flux of ferritinophagy. Elevated iron leads to NCOA4 degradation, promoting ferritin storage, while low iron increases NCOA4, boosting ferritinophagy to restore cellular iron. NCOA4’s influence extends beyond iron balance, impacting sensitivity to ferroptosis. Depleting NCOA4 reduces susceptibility to ferroptosis, whereas overexpression heightens sensitivity (Quiles del Rey and Mancias, 2019).

#### 2.2.5 VDAC

The regulation of ferroptosis is significantly influenced by voltage-dependent anion channel (VDAC), a protein found in mitochondria. VDAC facilitates the exchange of ions and metabolites between the cytoplasm and mitochondria. In the context of ferroptosis, VDAC’s role involves regulating iron transport into the mitochondria. This regulation is crucial, as an accumulation of iron within the mitochondria promotes ROS generation, triggering lipid peroxidation and instigating ferroptosis (DeHart et al., 2018).

### 2.3 Lipid metabolism

#### 2.3.1 ACSL4

ACSL4, belonging to the family of long-chain fatty acyl CoA synthetases, emerges as a crucial participant in ferroptosis. ACSL4 specifically favors 20-carbon polyunsaturated fatty acids (PUFAs) like arachidonic acid (AA) and adrenaline (ADA). Through catalyzing the conversion of these PUFAs into their CoA esters, ACSL4 actively contributes to the generation of lipid peroxides (LPO), thereby promoting the occurrence of ferroptosis. The gene governing ACSL4 is influenced by the oncoprotein YAP, suggesting a potential regulatory pathway impacting ACSL4 expression levels (Wu et al., 2019). Extensive research has emphasized the clinical significance of ACSL4 across diverse cancers, serving dual roles as a predictive and prognostic biomarker. In cancer therapy, directing interventions at ACSL4 has exhibited efficacy in triggering ferroptosis and impeding cell viability, presenting itself as a promising therapeutic approach. Moreover, ACSL4 is implicated in conferring resistance to specific drugs, such as sorafenib, particularly in hepatocellular carcinoma (Lin-Lin et al., 2021). Taken together, the distinctive involvement of ACSL4 in ferroptosis, coupled with its relevance to cancer, positions it as a prospective target for therapeutic interventions (Figure 1) (Yang et al., 2022).

#### 2.3.2 LPCATs

Lysophosphatidylcholine acyltransferase (LPCAT) is a vital enzyme crucial for maintaining the balance of phosphatidylcholine (PC). Particularly noteworthy is LPCAT3, which is widely distributed in the human liver, intestines, and adipocytes. LPCAT3 is involved in various aspects of lipid metabolism, such as influencing intestinal lipid absorption, regulating lipoprotein secretion, and contributing to liver fat synthesis, thereby impacting systemic lipid homeostasis. Research indicates that suppressing LPCAT3 can decrease polyunsaturated phospholipid levels and downregulate fat-production genes. Moreover, recent studies have linked LPCAT3 to ferroptosis, revealing that inhibiting LPCAT3 expression can impede ferroptosis. LPCAT3 actively promotes the esterification of PUFAs into phospholipids, serving as the building blocks for lipid peroxidation and supporting the occurrence of ferroptosis (Figure 1) (Hashidate-Yoshida et al., 2015; Li and Li, 2020).

#### 2.3.3 ALOXs

ALOX15, part of the ALOX enzyme family, is a crucial nonheme iron-containing dioxygenase involved in introducing oxygen into PUFAs. Among the six human *ALOX* genes, *ALOX15* plays a central role in translating oxidative stress into lipid peroxidation and ferroptosis. It forms a complex with phosphatidylethanolamine binding protein 1 (PEBP1), generating lipid peroxides and regulating ferroptotic cell death in various cell types. Unlike some other ALOX members, ALOX15 seems to be a core mediator in ferroptosis, influenced by diverse pathways and cellular responses. It responds to TP53-mediated signaling in lung cancer cells, and its activity is affected by exosomal miR-522 secretion from cancer-associated fibroblasts, contributing to ferroptosis resistance in gastric cancer cells. Notably, in situations where ferroptosis is initiated by the direct inhibition of GPX4, ALOX15’s role may be dispensable, as demonstrated by experiments involving the conditional knockout of GPX4. In these cases, ferroptotic damage can be reversed by radical-trapping antioxidants but not by depleting ALOX15 (Lin et al., 2021).

## 3 Overview of PI3K/Akt signaling

**FIGURE 2:**
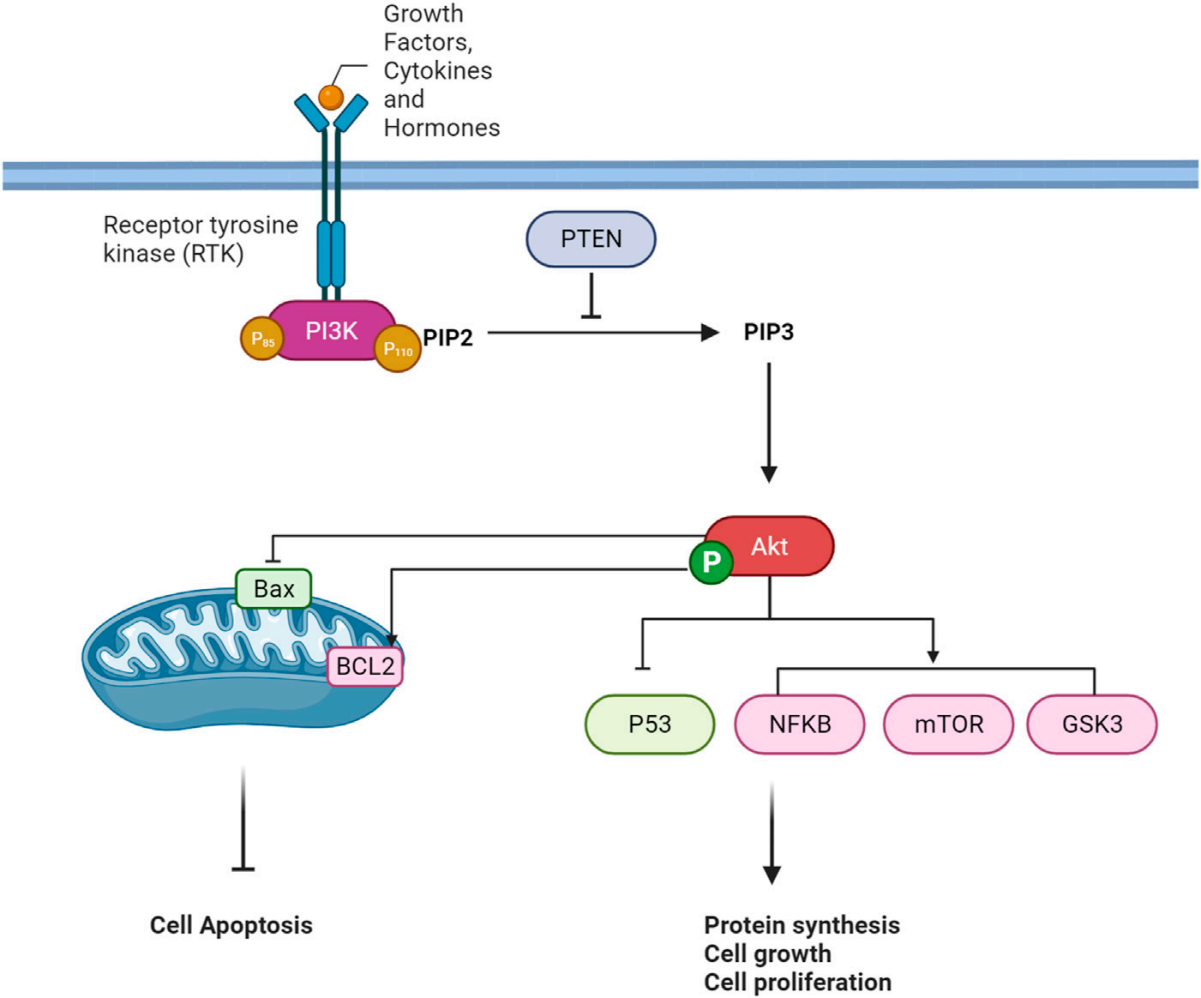
An overview of PI3K/Akt Pathway Cascade.

The PI3K/Akt pathway is a crucial intracellular signaling pathway that is a key element in regulating various cellular processes, including cell survival, growth, proliferation, and metabolism. The pathway is typically activated in response to extracellular signals such as growth factors, cytokines, and hormones. A quick overview of this pathway is provided in Figure 2.

### 3.1 Extracellular signals

Extracellular signaling pathways represent a complex and vital regulatory system that orchestrates cellular responses to various environmental stimuli. In cellular communication, the recognition and interpretation of extracellular signals play a fundamental function in determining cellular behavior (Hastings et al., 2019). Growth factors, cytokines, and hormones emerge as key signaling molecules that elicit cellular responses, regulating cell growth, differentiation, survival, and metabolism (Miricescu et al., 2021; Feng et al., 2022). The interaction of these signaling molecules with their corresponding receptors, particularly receptor tyrosine kinases (RTKs), initiates intracellular signaling cascades (Chen et al., 2017; Jiang and Ji, 2019). Ligand-receptor binding induces conformational changes in the receptors, leading to autophosphorylation and activation of downstream signaling pathways. In the context of the phosphoinositide 3-kinase/Akt (PI3K/Akt) pathway, a prototypical signaling cascade, activation is initiated by extracellular signals, including growth factors and hormones. This activation culminates in the generation of second messengers, such as phosphatidylinositol 3,4,5-trisphosphate (PIP3), and subsequent activation of the serine/threonine kinase Akt (Truebestein et al., 2021).

### 3.2 Receptor tyrosine kinases (RTKs) activation

When ligands attach to the extracellular portion of RTKs, it induces a structural alteration in the receptor, prompting the autophosphorylation of tyrosine residues within the RTK’s intracellular domain (Karl and Hristova, 2021). These phosphorylated tyrosine residues on the activated RTK act as sites for the binding of proteins containing Src homology 2 (SH2) domains, including regulatory subunits of phosphoinositide 3-kinase (PI3K), such as p85. PI3K is a dual-unit enzyme composed of a regulatory subunit (p85) and a catalytic subunit (p110). The interaction between p85 and phosphorylated tyrosine residues on the activated RTK alleviates inhibitory constraints on the catalytic function of p110 (Fox et al., 2020; Wang et al., 2020; Tabnak et al., 2023). Consequently, this activation leads to the conversion of phosphatidylinositol 4,5-bisphosphate (PIP2) to phosphatidylinositol 3,4,5-trisphosphate (PIP3) within the cell membrane. PIP3 functions as a secondary messenger and serves as a docking site for proteins containing pleckstrin homology (PH) domains, such as Akt (also referred to as protein kinase B or PKB) (Tariq and Luikart, 2021). The binding of Akt to PIP3 translocates it to the plasma membrane, where it undergoes phosphorylation and activation by phosphoinositide-dependent kinase 1 (PDK1) and mammalian target of rapamycin complex 2 (mTORC2) (Fu and Hall, 2020). Once fully activated, Akt can phosphorylate various downstream targets implicated in cell survival, growth, and metabolism (Hoxhaj and Manning, 2020).

### 3.3 PTEN

PTEN serves as a critical regulator in the PI3K/AKT signaling pathway by negatively modulating the levels of phosphatidylinositol 3,4,5-trisphosphate (PIP3). Acting as a tumor suppressor, PTEN counteracts the activity of PI3K by dephosphorylating PIP3 back to phosphatidylinositol 4,5-bisphosphate (PIP2) in the cell membrane. This action prevents the excessive accumulation of PIP3, inhibiting the recruitment and activation of downstream signaling proteins such as AKT. PTEN’s role establishes a crucial negative feedback loop in the pathway, contributing to the regulation of cell growth, survival, and metabolism. Dysregulation or loss of PTEN function is associated with elevated PIP3 levels, leading to uncontrolled cellular proliferation and is often implicated in cancer development (Jang et al., 2021).

### 3.4 p53

AKT, a serine/threonine kinase, indirectly regulates the tumor suppressor protein p53 through multiple cellular mechanisms. Under physiological conditions, AKT phosphorylates and inactivates MDM2, a negative regulator of p53, preventing the degradation of p53 and allowing its accumulation. Additionally, AKT-mediated phosphorylation of MDM2 enhances its stability, further inhibiting p53 activation. The crosstalk between the AKT and p53 pathways is vital in modulating the cellular response to stress and DNA damage. In conditions where AKT is highly active, it can suppress the pro-apoptotic functions of p53, promoting cell survival (Chen et al., 2019; Han et al., 2019).

### 3.5 NFκB

The serine/threonine kinase Akt assumes a crucial role in overseeing diverse cellular functions such as cell survival, proliferation, and motility. Its activation is initiated by lipid products stemming from PI3K. Following activation, Akt significantly influences the NFκB pathway, a cluster of transcription factors governing inflammation and immune responses. Akt triggers NFκB activation by stimulating the IκB kinase (IKK) complex through the phosphorylation of IKKα at threonine 23. This activation leads to the phosphorylation and subsequent degradation of the inhibitor of κB (IκB), facilitating NFκB migration into the nucleus for the regulation of gene transcription. Additionally, Akt phosphorylates the p65 subunit of NFκB at serine 534, intensifying NFκB-mediated transcription. The interaction between Akt and NFκB is linked to oncogenic transformation, and the reliance of PI3K/Akt on NFκB activity suggests potential therapeutic implications for cancer treatment (Bai et al., 2009).

### 3.6 mTOR

Akt, a serine/threonine kinase, tightly governs the signaling pathways of both mTORC1 (mammalian target of rapamycin complex 1) and mTORC2 (mammalian target of rapamycin complex 2). The activation of Akt, spurred by growth factors, involves site-specific phosphorylation. Upon activation, Akt significantly impacts mTORC1 by suppressing the tuberous sclerosis complex (TSC), leading to the activation of Rheb, an essential activator of mTORC1. Phosphorylation of TSC2 by Akt alleviates its inhibitory effect on Rheb, consequently promoting mTORC1 activity. Furthermore, Akt directly influences mTORC1 by phosphorylating its downstream targets (Tian et al., 2020b; Shams et al., 2021). While mTORC1 predominantly regulates processes such as protein synthesis and cell growth, mTORC2 is involved in the phosphorylation and activation of Akt itself. Akt’s reciprocal interaction with mTORC2 contributes to sustained Akt activation, amplifying its influence on cellular processes like cell survival and metabolism (Fu and Hall, 2020). In essence, Akt serves as a central regulator, linking growth factor signals to mTORC1 and mTORC2, thereby orchestrating a diverse network of cellular responses related to growth, proliferation, and survival (Memmott and Dennis, 2009).

### 3.7 GSK3

Through phosphorylation, Akt plays a regulatory role in glycogen synthase kinase 3 (GSK3). When activated by extracellular signals like growth factors or insulin, Akt targets specific serine residues on GSK3, Serine 9 for GSK3β, and Serine 21 for GSK3α. This phosphorylation event is essential in modulating cellular processes by inhibiting GSK3 activity. GSK3, a serine/threonine kinase, is implicated in various cellular functions, encompassing glycogen metabolism and cell cycle regulation. The inhibitory action of Akt on GSK3 contributes to cell survival and growth, enabling the activation of downstream pathways crucial for these essential cellular processes (Grabinski and Kanaan, 2016). This regulation, contingent on phosphorylation, is a critical element within the signaling network that orchestrates cellular responses to external stimuli.

## 4 PI3K/Akt pathway regulates key molecules of ferroptosis

The regulation of the PI3K/Akt-Ferroptosis axis involves a complex synergy of molecular regulators and cellular processes. PI3K/Akt signaling pathway activation can affect diverse molecules and components involved in ferroptosis. This section shows that particular components of ferroptosis, such as NRF2, GPX4, SLC7A11, iron, and lipid metabolism regulators, are influenced by downstream PI3K/Akt signaling pathway targets.

### 4.1 NRF2 effectors

#### 4.1.1 Protein kinase B (AKT)

**FIGURE 3:**
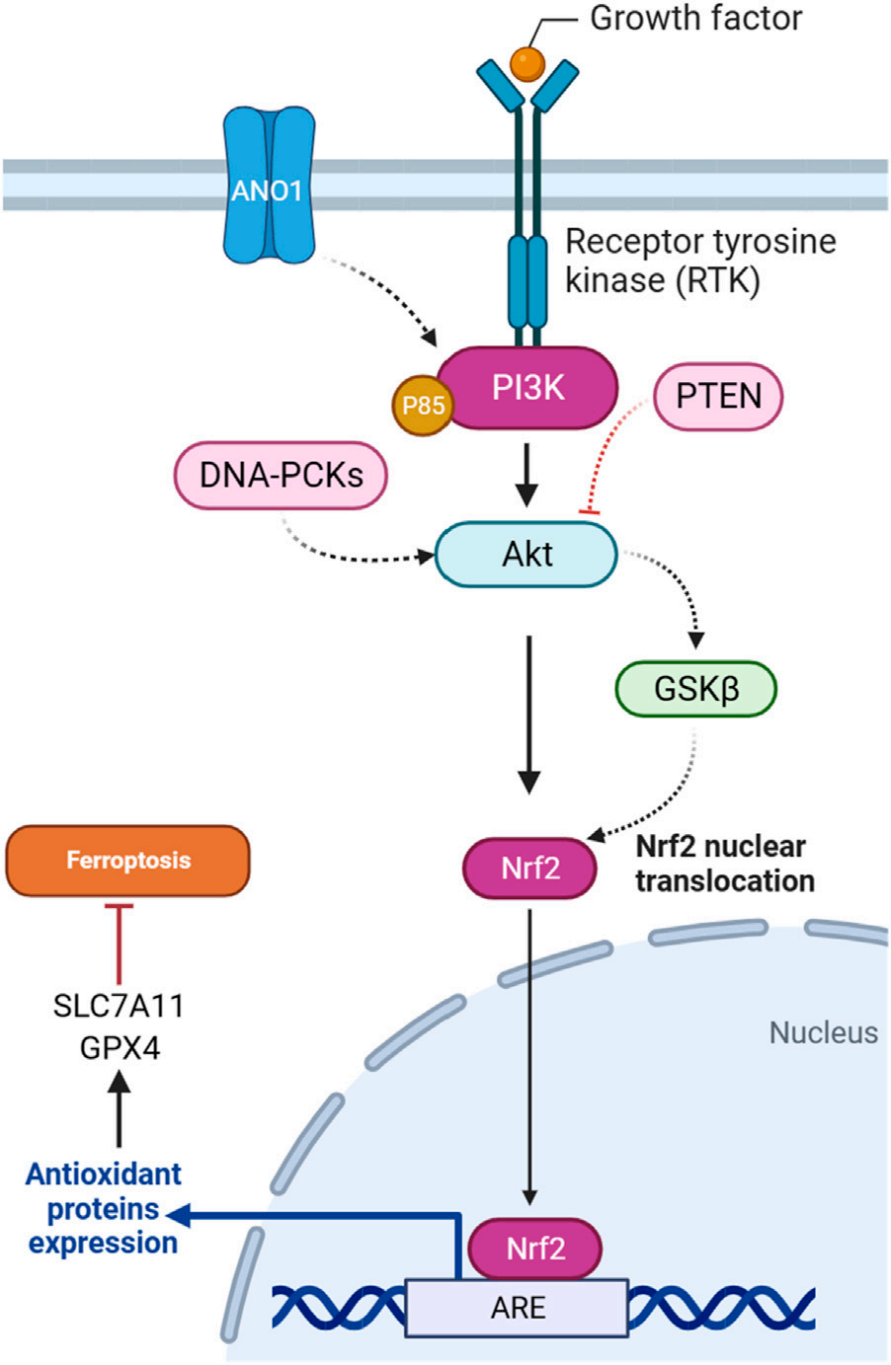
PI3K/Akt pathway-mediated NRF2 regulation inhibits ferroptosis.

In gliomas harboring pathogenic *IDH1* mutations, the activation of the PI3K/AKT pathway is implicated in tumor progression and treatment response. It has been demonstrated that heightened AKT activity is associated with enhanced cell survival and resistance to ferroptotic cell death in *IDH*-mutated glioma cells. The underlying mechanism involves AKT-mediated suppression of the Nrf2 E3 ligase bTrCP, resulting in the constant activation of Nrf2-driven gene transcription (Figure 3). This activation of the Nrf2/antioxidant pathway, renowned for its cytoprotective functions, is promoted by AKT in *IDH*-mutated glioma cells. The consequent increase in Nrf2-driven transcription, coupled with the decrease in pathways related to ferroptosis, indicates an important role for the AKT/Nrf2 axis in impeding ferroptosis in glioma. Thus, targeting the AKT/Nrf2 pathway has a therapeutic potential to sensitize *IDH*-mutated glioma cells to ferroptosis, offering novel strategies for treating these malignancies (Liu et al., 2023a).

#### 4.1.2 GSK3-β

There is a regulatory relationship between GSK3β and NRF2 in the context of ferroptosis induction in cisplatin-resistant gastric cancer cells. NRF2 is subject to regulation by GSK3β, particularly within the AKT/GSK3β/β-TrCP phosphorylation-dependent ubiquitination system. YJD (Yin-Ju-Di), a traditional Chinese medicine, is introduced as a potential therapeutic agent that inhibits NRF2 expression through the AKT/GSK3β pathway in gastric cancer (Figure 3). The use of an AKT activator, SC79, counteracts the effects of YJD, suggesting that YJD induces ferroptosis by modulating the NRF2/GPX4 axis via the AKT/GSK3β pathway (Huang et al., 2024). Furthermore, the presence of sodium butyrate (NaB), a compound produced through the fermentation of dietary fiber in the colon, induces the activation of GSK3β in colorectal cancer. This activation, in turn, leads to the phosphorylation of NRF2. Subsequently, phosphorylated NRF2 undergoes degradation through the ubiquitin-proteasome pathway facilitated by the protein β-TRCP1. As NRF2 degradation occurs, the downstream expression of *SLC7A11*, a crucial component of the cellular antioxidant defense system, is reduced. The downregulation of *SLC7A11*, resulting from NRF2 degradation induced by GSK3β, may render cells more susceptible to ferroptosis, as the cellular antioxidant capacity is compromised (Bian et al., 2023).

#### 4.1.3 PTEN

*PTEN*, as a tumor suppressor gene, is involved in regulating ferroptosis through the PI3K/Akt pathway. A study explored how PTEN loss influences ferroptosis susceptibility by modulating the cystine-xCT-GSH-GPX4 axis. PTEN loss is associated with increased expression of the *SLC7A11*, leading to higher cystine import, elevated GSH synthesis, and resistance to ferroptosis in many cancer cell lines. The mechanism operates through PTEN, which negatively regulates AKT (Figure 3). This, in turn, activates GSK3β, ultimately leading to a reduction in NRF2 levels. NRF2 is a transcription factor that promotes *xCT* expression. The findings suggest a new tumor-suppressive role of PTEN involving the modulation of cysteine metabolism, redox balance, and ferroptosis susceptibility (Cahuzac et al., 2023).

#### 4.1.4 DNA-PKc

The DNA-dependent protein kinase catalytic subunit (DNA-PKc) plays a key role as a critical enzyme in mending DNA double-strand breaks (DSBs) via the non-homologous end joining (NHEJ) pathway. Activation and detachment from DNA ends are contingent upon autophosphorylation at specific sites. The interaction of DNA-PKc with Ku proteins significantly contributes to the identification and binding of DNA ends. Beyond its primary involvement in DNA repair mechanisms, DNA-PKc influences cell cycle regulation, particularly impacting the G2/M checkpoint. Its application in cancer therapy to potentiate the efficacy of radiotherapy is an area of active exploration. Moreover, DNA-PKc is implicated in various cellular processes, such as telomere maintenance and the response to replication stress, maintaining genome stability (Yue et al., 2020). It has been suggested that DNA-PKc influences ferroptosis in addition to autophagy in osteosarcoma cells. Downregulation of DNA-PKcs is associated with reduced phosphorylation of AKT and affects the PI3K/AKT/NRF2 pathway (Figure 3), which regulates the antioxidant enzyme GPX4. It has been proposed that the decrease in DNA-PKcs, AKT, NRF2, and GPX4 levels contributes to the promotion of ferroptosis. The observed increase in lipid ROS and malondialdehyde (MDA) further supports the idea that DNA-PKcs downregulation enhances ferroptosis in osteosarcoma cells (Zhang Y. et al., 2023).

#### 4.1.5 ANO1

ANO1, also known as TMEM16A, functions as a calcium-activated chloride channel and is implicated in various malignant tumors, influencing their progression, metastasis, proliferation, and ability to resist apoptosis. ANO1’s involvement in impeding tumor apoptosis and promoting immune evasion is spotlighted (Guo et al., 2022). The ANO1-PI3K/AKT/NRF2 axis plays a crucial role in gastrointestinal (GI) cancers (Figure 3). ANO1, a calcium-activated chloride channel, regulates ferroptosis through this signaling pathway. ANO1 knockdown inhibits PI3K/AKT signaling, promotes ferroptosis, and reduces NRF2-associated ferroptosis resistance. Conversely, *ANO1* overexpression activates PI3K/AKT, leading to ferroptosis suppression. This ferroptosis modulation influences cancer-associated fibroblast (CAF) recruitment and TGF-β production, contributing to immunotherapeutic resistance in GI cancer. Targeting the ANO1-PI3K/AKT/NRF2 axis emerges as a potential strategy to enhance the efficacy of immunotherapy in GI cancer (Jiang F. et al., 2023).

### 4.2 SLC7A11 effectors

#### 4.2.1 STAT3 transcription factors

A study provided evidence that the combination of zerumbone (Zer) and gefitinib (Gef) in lung cancer treatment downregulates p-Akt and p-STAT3, indicating the inhibition of the AKT/STAT3 signaling axis. Additionally, the research demonstrates that the expression levels of *SLC7A11*, a component of the cystine/glutamate antiporter system Xc^−^, were decreased in tumor tissues following treatment with Zer and Gef. The AKT/STAT3 axis is implicated in various cellular processes, including cell survival and proliferation. The downregulation of p-Akt and p-STAT3 suggests that the combination treatment interferes with these signaling pathways, potentially influencing downstream targets such as SLC7A11 and inducing ferroptosis in lung cancer cells (Zhao et al., 2019; Wang J-G. et al., 2023).

#### 4.2.2 Hypoxia

In many human tumors, the PI3K/Akt signaling pathway is constantly active. Inhibiting this pathway reduces the activation of HIF-1α and restores the ability of cells to undergo apoptosis under low-oxygen conditions. Targeting PI3K/Akt also makes cancer cells more sensitive to apoptosis-inducing treatments (Kilic-Eren et al., 2013). The researchers investigated the reciprocal action between hypoxia, the PI3K/AKT/HIF-1α pathway, and ferroptosis in glioma cells. They observed that hypoxia-activated the PI3K/AKT pathway, leading to the stabilization of HIF-1α. This activation, in turn, upregulated the expression of *SLC7A11*. Elevated SLC7A11 levels under hypoxia were associated with increased resistance to ferroptosis. Further results demonstrated that inhibiting HIF-1α with PX-478 reversed ferroptosis resistance by downregulating *SLC7A11* expression. Moreover, the combination of PX-478 with the ferroptosis-inducing agent SAS had a synergistic anticancer effect in both *in vitro* and *in vivo* models, suggesting a potential therapeutic strategy for sensitizing glioma cells to ferroptosis through targeting the PI3K/AKT/HIF-1α/SLC7A11 axis. These findings shed light on the molecular mechanisms underlying the regulation of ferroptosis in glioma cells under hypoxic conditions, offering insights into novel therapeutic interventions for glioma treatment (Sun et al., 2022).

#### 4.2.3 CCT3/Akt/SLC7A11

Chaperonin-containing TCP1 (CCT) is a eukaryotic chaperone complex crucial for cell survival, recognizing various substrate protein motifs. CCT3, a major subunit, plays a fundamental role in protein folding, with its gene located on chromosome 1. CCT3 is involved in regulating signaling pathways like STAT3, Wnt, and PI3K-AKT, impacting tumor cell proliferation, differentiation, and apoptosis (Liu W. et al., 2022). The protein CCT3 plays a role in the ferroptosis of lung adenocarcinoma cells. This process involves the transcriptional activation of *SLC7A11* gene by CCT3. Additionally, CCT3 influences the Akt signaling pathway, which is associated with various malignant features, including cell growth and metastasis. In this context, Akt activation by CCT3 contributes to the suppression of ferroptosis (Wang K. et al., 2022).

#### 4.2.4 ATP binding molecules

ABCC5, also known as multidrug resistance-associated protein 5 (MRP5), is a member of the ATP-binding cassette (ABC) transporter family. ABC transporters are ATP-powered pumps involved in transporting various molecules across cell membranes. Additionally, mutations in *ABC* genes can lead to genetic diseases, and certain ABC transporters are implicated in drug resistance in cancer cells, such as in hepatocellular carcinoma (HCC) (Qiu et al., 2021). A study elucidated the critical role of ABCC5 in HCC sorafenib resistance cells, with a multifaceted impact on ferroptosis regulation. Sorafenib treatment activates the PI3K/AKT pathway, leading to increased expression of *NRF2* and *ABCC5*. ABCC5, in turn, physically interacts with SLC7A11. This interaction suppresses lipid peroxidation and mitochondrial damage induced by sorafenib, contributing to acquired resistance. The study proposes an axis involving NRF2, ABCC5, and SLC7A11, suggesting complex coordination in cellular defense mechanisms. Inhibition of *ABCC5* expression enhances the anti-cancer activity of sorafenib, underscoring the potential therapeutic significance of targeting ABCC5 to overcome sorafenib resistance in HCC. The findings accentuate the PI3K/AKT pathway as a key upstream regulator, orchestrating the molecular events leading to ABCC5-mediated resistance and altered ferroptotic response in HCC cells treated with sorafenib (Huang et al., 2021). The regulatory mechanisms underlying the protection of gastric cancer cells from ferroptosis through ATP-binding cassette sub-family D member 1 (ABCD1) have been investigated. The proposed pathway involves the activation of the PI3K/Akt/mTOR/HIF1a signaling cascade in response to serotonin (5-HT) stimulation through HTR2B. HIF1α, a transcription factor downstream of this pathway, translocates to the nucleus and directly binds to the promoter region of the *ABCD1* gene. ABCD1 is implicated in lipid metabolism and ferroptosis. The results suggest that the transcriptional regulation of *ABCD1* by HIF1α constitutes a protective mechanism, suppressing ferroptosis and promoting cell survival under metabolic stress conditions.

### 4.3 GPX4 effectors

#### 4.3.1 mTORC1

**FIGURE 4:**
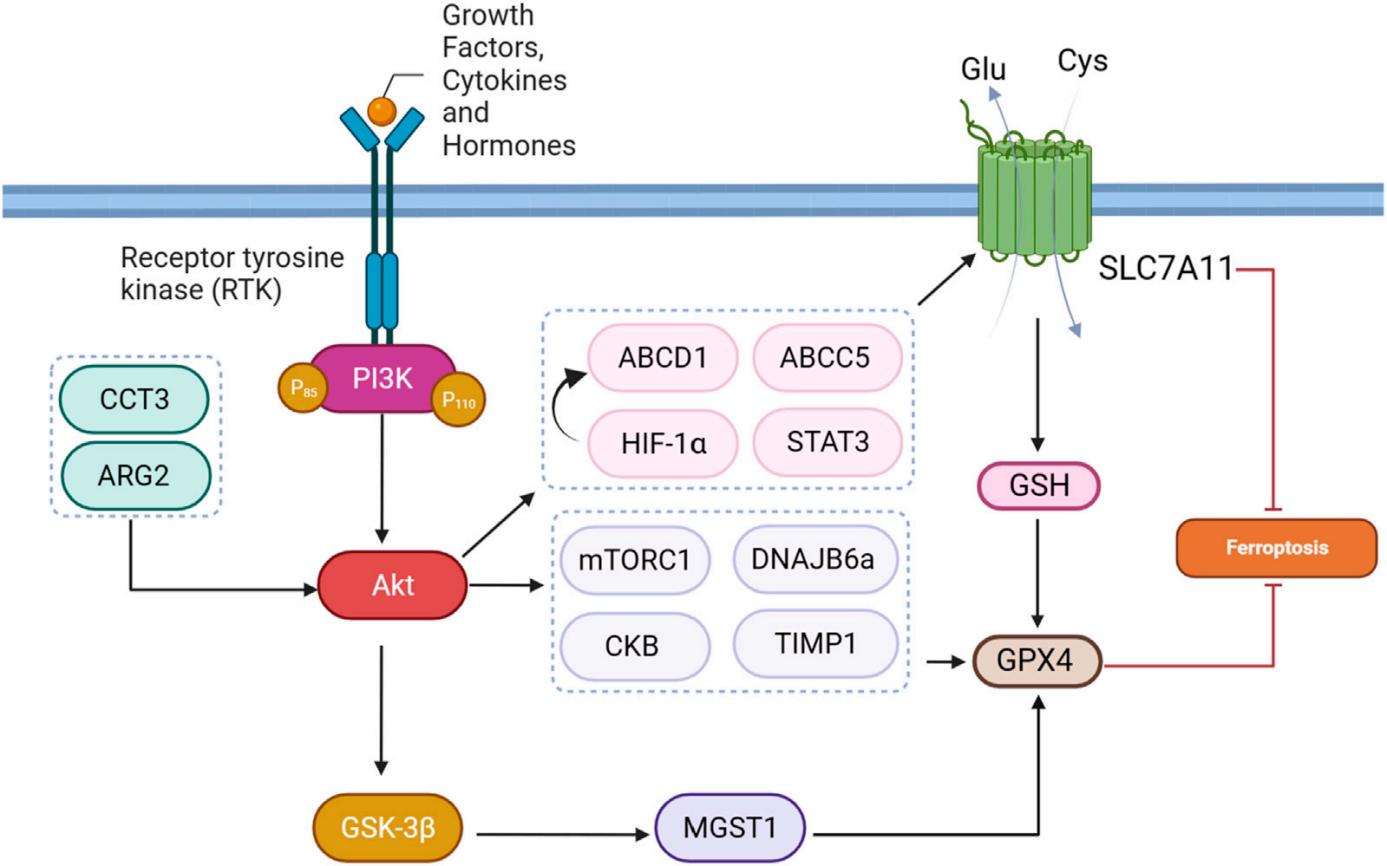
PI3K/Akt pathway-mediated GPX4 and SLC7A11 regulation inhibits ferroptosis.

mTORC1, a downstream effector of the PI3K-AKT pathway, is a master ferroptosis regulator. Acting as a protective “shield” against ferroptosis, mTORC1 is implicated in inhibiting this process in cancer cells through multiple mechanisms. One key mechanism involves mTORC1-mediated promotion of GPX4 protein synthesis, a crucial enzyme that guards against ferroptosis by preventing lipid peroxidation (Lei et al., 2021). Wang et al. found that sodium butyrate (NaB) treatment exerts regulatory effects on ferroptosis through a multi-step mechanism in many cencer cells. Initially, NaB induces the inhibition of mTORC1 activity, evidenced by a reduction in the phosphorylation levels of downstream targets such as S6K, 4EBP1, and S6. Importantly, mTORC1 activation has been linked to the promotion of GPX4 protein synthesis (Figure 4). Consequently, NaB-induced mTORC1 inactivation correlates with a suppression of GPX4 synthesis. Therefore, NaB, through its modulation of the mTORC1 pathway, plays a role in regulating ferroptosis by impacting the expression of GPX4, ultimately influencing cellular susceptibility to ferroptotic stimuli (Wang G. et al., 2023). AKT/mTORC1/4EBP1 signaling pathway exerts a key function in mediating the effects of fatostatin on glioblastoma multiforme (GBM) cells. Specifically, the inhibition of this pathway by fatostatin appears to orchestrate a cascade of events leading to decreased levels of GPX4 protein and the subsequent induction of ferroptosis in GBM cells (Cai et al., 2023).

#### 4.3.2 Creatine kinase B

Creatine kinase B (CKB) is an enzyme primarily present in the brain and smooth muscle tissues, where it serves a crucial role in cellular energy metabolism. Its primary function involves mediating the transfer of phosphate groups between creatine and adenosine triphosphate (ATP), thereby contributing to cellular energy requirements. Especially in tissues with variable energy demands, such as the brain, CKB plays a vital role in maintaining energy homeostasis. In HCC, insulin-like growth factor 1 receptor (IGF1R) signaling activates AKT, leading to the phosphorylation of CKB at a specific site (T133). This phosphorylation event results in a dual role for CKB. In its conventional function, CKB continues to support cellular energy metabolism by facilitating phosphate transfer between creatine and ATP. Simultaneously, in its “moonlighting” function, phosphorylated CKB interacts with GPX4 (Figure 4). This interaction triggers the phosphorylation of GPX4 at S104. The phosphorylated GPX4, positioned adjacent to the chaperone-mediated autophagy (CMA) target motif, hinders the binding of HSC70 to GPX4, thereby preventing CMA-mediated GPX4 degradation. This molecular interrelation sheds light on the dual functionality of CKB, emphasizing its involvement in both energy metabolism and the regulation of ferroptosis in the context of HCC and potentially other cellular processes (Wu et al., 2023).

#### 4.3.3 AKT induced m^6^A modification

The dynamic regulation of target genes occurs through N6-Methyladenosine (m^6^A) modification across various stages of RNA metabolism (Chen S. et al., 2022). This process encompasses key actors: writers, erasers, and readers, each fulfilling unique functions. The METTL3-METTL14 complex, classified as writers, introduces m^6^A to precise adenosines within RNA molecules. Conversely, erasers like FTO and ALKBH5 eliminate m^6^A modifications, establishing a reversible aspect of the process. Readers, exemplified by YTH-containing proteins and hnRNPs, decode the information embedded in m^6^A-modified RNA, thereby shaping the destiny of target genes. This mutual influence reflects the complexity of m^6^A modification in orchestrating gene expression dynamics through its multifaceted regulatory machinery (Wang S. et al., 2022). Inhibition of the AKT pathway using the MK2206 inhibitor was found to have a profound impact on the occurrence of ferroptosis through the modulation of m^6^A modification in colorectal cancer. The inhibition of AKT led to the downregulation of *FTO*, resulting in increased levels of m^6^A modification on RNA. Notably, the *GPX4* gene, crucial for preventing ferroptosis, was identified as a target of m^6^A modification. The m^6^A modification at the 193 site on *GPX4* mRNA facilitated the binding of the m^6^A reader protein YTHDF2, leading to the degradation of *GPX4* mRNA and subsequent reduction in GPX4 protein levels. With decreased GPX4 levels, the cell’s defense against lipid peroxidation was compromised, ultimately inducing ferroptosis. This unveils a novel regulatory axis wherein AKT pathway inhibition influences ferroptosis through the m^6^A modification of GPX4, shedding light on a previously unrecognized mechanism in cell survival and death processes (Zhang G. et al., 2023).

#### 4.3.4 MGST1

MGST1, or microsomal glutathione S-transferase 1, emerges as a critical player in conferring resistance to ferroptosis in cancer cells, particularly illustrated in the context of SGC7901 gastric cancer cells. Ferroptosis can be induced by various stimuli, including drugs like sorafenib, erastin, and RSL3. In these cancer cells, exposure to ferroptosis inducers leads to a reduction in MGST1 expression at both protein and mRNA levels. Notably, the overexpression of MGST1 counteracts ferroptosis, as evidenced by increased cell viability and a reduction in ferroptotic markers such as MDA, iron, glutathione GSH, and ROS. Mechanistically, MGST1 achieves ferroptosis resistance through the activation of the Akt/GSK-3β pathway (Figure 4). The Akt inhibitor MK-2206 effectively reverses this protective effect, indicating the involvement of the Akt/GSK-3β pathway in mediating the resistance to ferroptosis conferred by MGST1. Overall, MGST1 stands out as a key regulator, modulating the delicate balance between cell survival and ferroptotic cell death in GI cancer cells through extensive molecular pathways (Peng and Peng, 2023). The MGST1/AKT/GSK-3β axis tightly regulates ferroptosis in gastric cancer cells. Overexpression of *MGST1* activates Akt/GSK-3β signaling, resulting in the downregulation of *GPX4*, a key ferroptosis regulator. This activation compromises GPX4’s protective role against lipid peroxidation, promoting ferroptosis resistance. Treatment with the Akt inhibitor MK-2206 reverses these effects, inhibiting Akt phosphorylation, restoring GPX4 levels, and attenuating the inhibitory impact of MGST1 on ferroptosis. In summary, the MGST1/AKT/GSK-3β axis modulates ferroptosis by regulating GPX4 levels and activity in gastric cancer cells (Li Y. et al., 2023).

#### 4.3.5 DNAJB6

DNAJB6, represented by its isoforms DNAJB6a and DNAJB6b, engages in a range of cellular activities and contributes to diverse health conditions, including cancer. It exhibits anti-tumor effects in breast and melanoma cancers by suppressing Wnt/β-catenin signaling, whereas it fosters aggressiveness in colon cancer by promoting cell adhesion and migration through interaction with HSP70 and uPAR, activating the MAPK pathway. The variable roles of DNAJB6 underscore its significance as a potential biomarker and therapeutic target in specific cancer contexts (Jiang et al., 2020). A study explored the impact of DNAJB6a in esophageal squamous cell carcinoma (ESCC), emphasizing its association with cancer-related processes, ferroptosis, AKT signaling, and GPX4 (Figure 4). Lower DNAJB6a levels in ESCC tissues was linked to lymph node metastasis. Lentivirus-induced DNAJB6a upregulation suppressed ESCC cell proliferation, migration, and invasion, suggesting a tumor-suppressive role. Notably, increased levels of DNAJB6a promoted oxidative stress, affecting lipid peroxidation and reducing GSH levels, indicating a potential involvement in ferroptosis. Altered mitochondrial structure and reduced levels of GPX4 and p-AKT were observed, implying modulation of ferroptosis and AKT signaling by DNAJB6a in ESCC cells (Jiang et al., 2020).

#### 4.3.6 TIMP1

Tissue inhibitor of metalloproteinases 1 (TIMP1) is a protein with a significant role in moderating the function of enzymes called matrix metalloproteinases (MMPs). MMPs are responsible for breaking down elements of the extracellular matrix, a crucial process in various physiological activities such as tissue remodeling and cell migration. TIMP1 functions as an inhibitor by regulating the activity of MMPs, thus impacting the equilibrium of extracellular matrix breakdown. TIMP1 plays a role in conferring resistance to ferroptosis in colorectal cancer cells. Specifically, TIMP1 overexpression induces sorafenib resistance, and its knockdown is associated with the inhibition of the PI3K/Akt pathway (Figure 4). The activation of the PI3K/Akt pathway, in turn, leads to increased expression of GPX4. Therefore, in this scenario, TIMP1-induced activation of the PI3K/Akt pathway and subsequent upregulation of GPX4 contribute to resistance against sorafenib-triggered ferroptosis in colorectal cancer cells (Wang L. et al., 2023).

#### 4.3.7 ARG2

Arginase 2 (ARG2) is a manganese-containing metalloenzyme that plays a crucial role in the urea cycle, converting L-arginine to L-ornithine and urea. ARG2 is primarily found in the mitochondria and is abundantly expressed in various tissues, including the kidney and prostate. In the context of cancer, ARG2 has been implicated in promoting tumorigenesis through its impact on signaling pathways. Notably, ARG2 activation has been linked to PI3K/AKT/mTOR signaling pathway. ARG2 activates mTORC1-S6 kinase 1 (S6K1), contributing to tumor progression (Niu et al., 2022). The Arg2/Akt/GPX4 signaling pathway plays a vital role in regulating ferroptosis (Figure 4). Sorafenib, known for inducing ferroptosis, reduces *Arg2* expression, accompanied by decreased Akt phosphorylation and GPX4 levels in melanoma cells. Overexpression of *Arg2* acts as a counterbalance to Sorafenib-induced ferroptosis by activating Akt and increasing GPX4, thereby inhibiting lipid peroxidation. This mechanism involves Arg2 impeding the Akt-GPX4 cascade, ultimately preventing ferroptotic cell death. Conversely, depletion or suppression of Arg2 activity enhances Sorafenib-induced ferroptosis, resulting in elevated lipid peroxidation, reduced cell viability, and impaired colony formation (Yu et al., 2022).

### 4.4 Lipid metabolism effectors

#### 4.4.1 SIRT3

**FIGURE 5:**
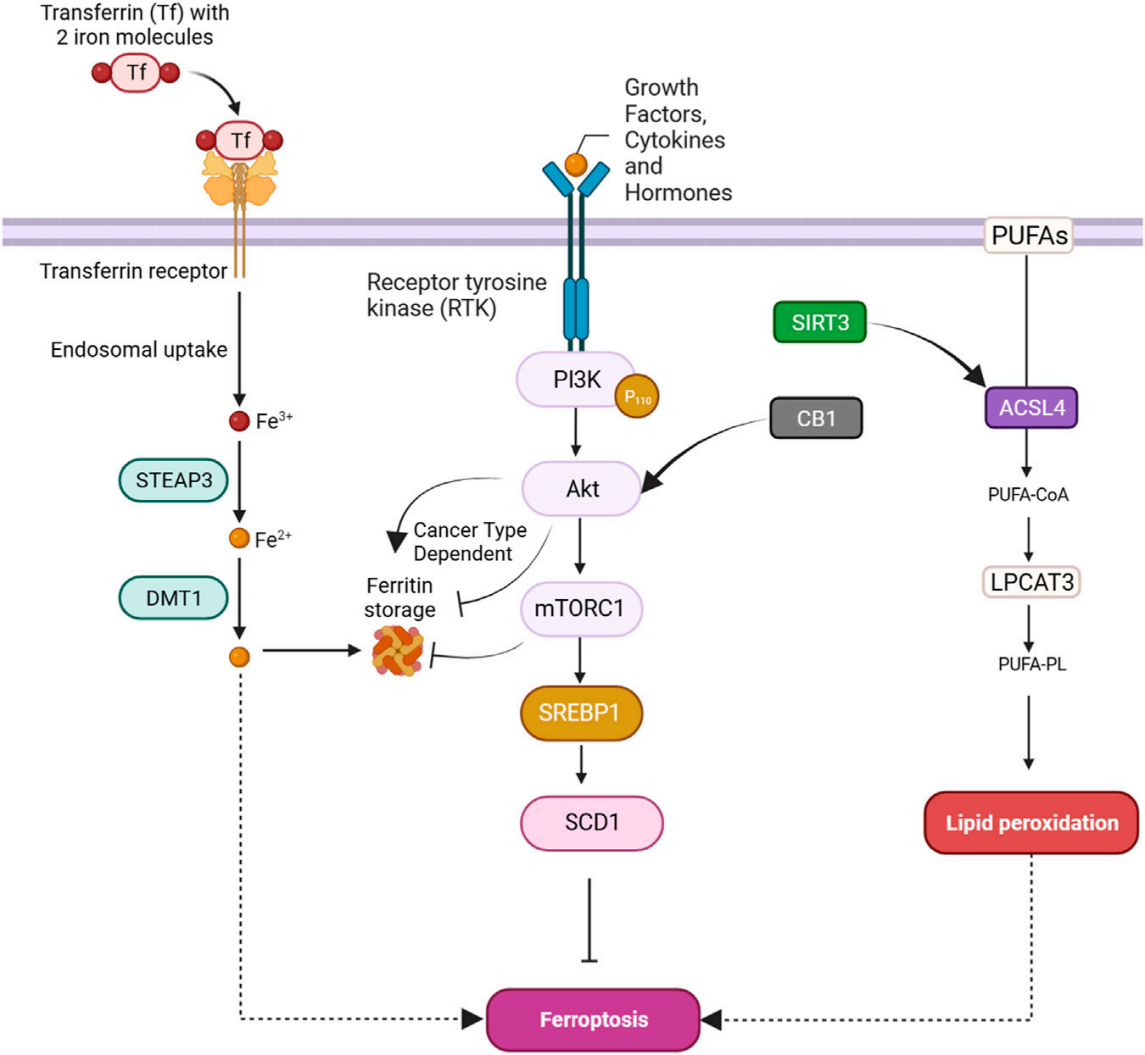
PI3K/Akt pathway-mediated lipid and iron metabolism regulation in ferroptosis.

SIRT3 is a member of the sirtuin family, functioning as a NAD^+^-dependent deacetylase primarily found in mitochondria. SIRT3 plays a complex role in cancer cells. It demonstrates both tumor-suppressive and oncogenic functions, influencing diverse aspects of cancer biology, including cell survival, metabolism, DNA repair, and oxidative stress response. The precise impact of SIRT3 in cancer appears to be context-dependent, varying across cancer types and stages (Liu et al., 2023b). In gallbladder cancer (GBC), *SIRT3* experiences substantial downregulation, influencing clinical outcomes. Its diverse functions extend to the regulation of mitochondrial metabolism, oxidative stress, DNA repair, and ferroptosis, positioning SIRT3 as a tumor suppressor in GBC. The tumor-suppressive role in GBC involves complex signaling pathways, particularly the AKT/PI3K pathway. *SIRT3* downregulation activates AKT, impacting cellular processes like ferroptosis, leading to changes in ATP production, redox balance, and lipid metabolism, with a notable impact on the expression of ACSL4, a crucial component in ferroptosis (Figure 5) (Liu et al., 2021).

#### 4.4.2 mTORC1

mTORC1 activates SREBP1, promoting the upregulation of stearoyl coenzyme A desaturase-1 (SCD1), a key enzyme in lipid metabolism. SCD1 protects cells from ferroptosis by converting SFAs to MUFAs, thereby inhibiting lipid peroxidation. Inhibition of mTORC1, combined with ferroptosis induction through GPX4 inhibition or erastin treatment, leads to tumor regression in mouse xenograft models. SCD1 is a vital regulator of cellular metabolism, influencing cell function by maintaining the equilibrium of fatty acids, and is implicated in cancer development. Targeting SCD1 is considered a potential therapeutic strategy for cancers resistant to ferroptosis induction, particularly those with mutations in the PI3K/AKT/mTOR pathway (Yi et al., 2020; Sen et al., 2023). In triple-negative breast cancer, the activation of CB1, as part of the CB1/Akt/mTOR/SCD1 signaling cascade, may impact ferroptosis (Figure 5). mTOR, a key regulator of cellular processes, including lipid metabolism, could potentially affect the lipid composition of cells, and SCD1 may contribute to alterations in lipid metabolism that influence cellular susceptibility to ferroptosis. Given that ferroptosis is marked by lipid peroxidation, changes in lipid composition influenced by CB1 activation might modulate the vulnerability of cells to ferroptosis cell death (Li P. et al., 2022).

### 4.5 FTH effectors

#### 4.5.1 AKT

FTH serves as a storage for cellular iron, maintaining iron balance within cells. When cellular processes, such as ferroptosis, necessitate adjustments in iron levels, FTH becomes a target for autophagic degradation. Autophagy, a cellular recycling mechanism, breaks down FTH, releasing stored iron into the labile iron pool in the cytoplasm (Tian et al., 2020a; Yan et al., 2023). In cisplatin-resistant ovarian cancer, a study investigated the relationship between AKT1, ferroptosis susceptibility, and autophagic degradation of FTH1. Cisplatin-resistant cells exhibited increased ferroptosis sensitivity, accompanied by increased levels of key molecules involved in the ferroptosis defense system. Notably, FTH1, a key player in iron metabolism, showed increased degradation in resistant cells, potentially contributing to enhanced ferroptosis sensitivity. This degradation was linked to elevated autophagy levels. Further analysis revealed that AKT1, a known autophagy inhibitor, was significantly reduced in cisplatin-resistant patients. Restoring AKT1 levels in resistant cells led to decreased autophagy, elevated FTH1 levels, and reduced ferroptosis-induced cell death (Figure 5) (Shi et al., 2023). In contrast, a study revealed that in Ewing sarcoma (ES), the Akt/FTH axis plays a role in inhibiting ferroptosis. The researchers investigated the molecular mechanisms underlying the inhibition of ferroptosis in ES cells. They observed that Akt, a key signaling molecule, is involved in the regulation of FTH. Akt activation was found to suppress ferroptosis by enhancing the stability of FTH, which, in turn, reduces the availability of free iron ions in the labile iron pool. This reduction in free iron ions is hypothesized to inhibit ferroptosis in ES cells (Fayzullina et al., 2023).

#### 4.5.2 mTORC1

mTOR is involved in regulating ferritin degradation through a process called ferritinophagy, which is a selective autophagic degradation of ferritin, the cellular iron storage protein. When mTOR is active, it suppresses autophagy initiation; however, its inhibition induces autophagy. In ferritinophagy, mTOR inhibition leads to the formation of autophagosomes that engulf ferritin. These autophagosomes fuse with lysosomes, forming autolysosomes where ferritin is degraded, releasing cellular iron (Figure 5). This process contributes to cellular iron homeostasis and is crucial for various cellular functions, including responses to oxidative stress and ferroptosis (Lei et al., 2021). Banxia Xiexin decoction (BXD) is used in Chinese traditional medicine against colorectal cancer. BXD constituents exhibited inhibitory effects on the proliferation and migration of colorectal cancer cells *in vitro*, concomitant with elevated levels of iron and ROS. The underlying mechanism involved the induction of ferritinophagy, resulting in ferritin degradation, coupled with the suppression of the PI3K/AKT/mTOR signaling pathway. These findings were substantiated through experimentation in a CRC xenograft model, demonstrating reduced tumor dimensions, heightened iron concentration, and augmented ROS levels (Wang et al., 2024a).

### 4.6 VDAC effectors

#### 4.6.1 CERK

Inhibition of ceramide kinase (CERK), a kinase associated with the generation of ceramide-1-phosphate (C1P), was explored in non-small cell lung cancer (NSCLC) cells harboring *KRAS* mutations. NSCLC cell lines with mutant *KRAS* exhibited significantly lower survival rates upon treatment with the CERK inhibitor NVP-231 compared to those with wild-type *KRAS*, indicating a specific vulnerability of *KRAS*-mutated cells to CERK inhibition. Mutant *KRAS* cells showed elevated CERK expression and C1P levels. Inhibition of CERK induced cell death through ferroptosis, as evidenced by increased lipid peroxidation and heightened sensitivity to ferroptosis inducers. CERK inhibition led to mitochondrial dysfunction, increased mitochondrial membrane potential, and modulation of VDAC–tubulin interactions. The effects of CERK inhibition were reversed by downregulation of VDAC1. Importantly, the research implicated the PI3K/AKT pathway, as CERK inhibition resulted in decreased AKT phosphorylation, and AKT inhibition mimicked the effects of CERK inhibition on cell survival, mitochondrial membrane potential, and VDAC-tubulin binding. Furthermore, combining the CERK inhibitor with cisplatin synergistically reduced cell survival in NSCLC cells with *KRAS* mutations. The findings underscore the critical role of CERK in regulating ferroptosis and suggest a potential therapeutic strategy for NSCLC with *KRAS* mutations by targeting CERK and modulating the AKT pathway (Vu et al., 2022).

## 5 Regulators of PI3K/Akt components

### 5.1 AMPK

The normal functioning of AMPK/Akt/mTOR signaling pathway cellular pathway encompasses regulating vital processes such as cell growth and survival in *KRAS*-mutant CRC cells. Osthole treatment results in the downregulation of phosphorylated AMPK, Akt, and mTOR, indicating an inhibition of this signaling cascade. This inhibition is associated with changes in the expression of genes related to ferroptosis, with an increase in AMPK-associated genes and a decrease in PI3K/Akt/mTOR-associated genes. Osthole-induced alterations manifest in reduced levels of GPX4, a negative regulator of ferroptosis, and an elevation in transferrin. The impact on ferroptosis sensitivity is evidenced by changes in cell viability, Annexin V staining, and cellular ferrous iron levels. Overall, osthole promotes ferroptosis in *KRAS*-mutant CRC cells, and this effect is attributed, at least in part, to the inhibition of the AMPK/Akt/mTOR signaling pathway, thereby influencing vital cellular processes and facilitating ferroptotic cell death (Zhou et al., 2023).

### 5.2 Transcription factors

#### 5.2.1 KLF2

Krüppel-like factor 2 (KLF2), a zinc finger transcription factor, plays a crucial role in regulating ferroptosis, in CRC cells. First, *KLF2* overexpression induces a significant upregulation of GPX4. Second, KLF2 exerts its influence on ferroptosis by inhibiting the phosphoinositide 3-kinase/protein kinase B (PI3K/AKT) signaling pathway. The inhibition of PI3K/AKT signaling is crucial, as this pathway is known to promote cell survival and inhibit programmed cell death. By suppressing this pathway, KLF2 removes a key obstacle to ferroptosis induction. Third, the collective effect of KLF2-induced GPX4 upregulation and PI3K/AKT pathway inhibition results in ferroptosis induction in CRC cells. This process involves the accumulation of iron and lipid peroxidation, ultimately leading to programmed cell death (Li J. et al., 2023).

#### 5.2.2 SREBP1

Jin et al. demonstrated a complex interaction between B7H3, the transcription factor SREBP2, and the AKT signaling pathway in the context of ferroptosis regulation in colorectal cancer cells. B7H3 knockdown enhances sensitivity to ferroptosis, as evidenced by morphological changes (e.g., cell shrinkage and volume reduction), decreased cell survival, and increased levels of malondialdehyde and iron in CRC cells. Mechanistically, B7H3 knockdown results in elevated expression of nuclear SREBP2 (n-SREBP2), a key regulator of cholesterol metabolism. The inhibitory effect of B7H3 on ferroptosis is mediated, at least in part, by its modulation of cholesterol metabolism through SREBP2. Moreover, their study implicated the AKT signaling pathway in this regulatory network, as B7H3 knockdown decreases AKT phosphorylation, and inhibition of AKT enhances n-SREBP2 expression. This suggests that B7H3 may regulate the AKT-SREBP2 axis to influence cholesterol metabolism and subsequently modulate the sensitivity of CRC cells to ferroptosis. These findings provide insights into the molecular mechanisms underlying the relationship between B7H3, SREBP2, and AKT signaling in ferroptosis regulation, offering potential therapeutic avenues for CRC treatment (Yu et al., 2022). In many cancer cells, mTORC1, a downstream component of the PI3K-AKT pathway, is responsible for ferroptosis resistance. Specifically, mTORC1, but not mTORC2, suppresses ferroptosis, and this effect is mediated by upregulating the transcription factor SREBP1. SREBP1, in turn, protects cells from ferroptosis by promoting the expression of SCD1, an enzyme involved in fatty acid desaturation. Thus, combining mTORC1 inhibition with ferroptosis induction could be a promising therapeutic strategy for cancers with PI3K-AKT pathway mutations, demonstrating significant tumor regression in mouse xenograft models (Battaglia et al., 2020).

### 5.3 Non-coding RNAs

MicroRNAs are small non-coding RNA molecules that play a crucial role in regulating gene expression. They can be involved in various cellular processes, including those related to inflammation, apoptosis, and immune response (Cui et al., 2022). MiR-21-5p exerts its inhibitory effect on ferroptosis HCC cells by modulating the Akt/mTOR signaling pathway, a regulatory axis critical for the progression of HCC. MiR-21-5p likely targets and downregulates key components of the Akt/mTOR pathway, such as AKT and mTOR themselves, as well as potentially influencing downstream effectors. This regulation disrupts the delicate balance necessary for ferroptosis induction. The characterization of ferroptosis inhibition encompasses the observed elevation in crucial anti-ferroptotic proteins, including GPX4, FTH1, and xCT, and a concurrent decrease in the levels of HO-1. Furthermore, the alterations in cellular levels of GSH, ROS, and Fe^2+^ contribute to the miR-21-5p-mediated ferroptosis inhibition (Hu et al., 2023). A2M-AS1, a long non-coding RNA, has been revealed to induce ferroptosis by influencing the activation of key signaling pathways in pancreatic cancer cells. In particular, the overexpression of A2M-AS1 significantly enhanced the activation of p38, a pathway associated with the induction of ferroptosis. Concurrently, A2M-AS1 upregulation demonstrated an inhibitory effect on the AKT/mTOR signaling pathways, which are known to impede the progression of ferroptosis. Conversely, silencing A2M-AS1 had the opposite effect on these pathways. Additionally, PCBP3, a member of the hnRNP family, showed similar regulatory effects on p38 and AKT/mTOR signaling pathways. Notably, the influence of *A2M-AS1* silencing on these pathways was effectively counteracted by the overexpression of PCBP3 (Qiu et al., 2022).

### 5.4 PAQR3

PAQR3, part of the progestin and adipoQ receptor superfamily, is a Golgi-resident protein with a seven-transmembrane domain structure. While previously known for its negative regulation of signaling pathways like Ras-Raf-Mek-Erk and PI3K-AKT, recent research indicates a novel role for PAQR3 as a positive mediator in autophagy initiation. It preferentially facilitates the formation of specific protein complexes associated with autophagy, contributing to cellular processes involved in the degradation and recycling of cellular components (Xu et al., 2016). PAQR3 inhibits ferroptosis in diffuse large B-cell lymphoma (DLBCL) cells by targeting the LDLR/PI3K/AKT pathway. Overexpression of PAQR3 reduces LDLR expression and dampens PI3K/AKT pathway activation. This inhibition decreases DLBCL cell viability and induces ferroptosis, characterized by elevated MDA, ROS, and Fe^2+^ levels and reduced GSH, GPX4, and SLC7A11 levels. The inhibitory effect on ferroptosis by PAQR3 is partially reversed by overexpressing LDLR. *In vivo*, OE-PAQR3 suppresses DLBCL tumor growth and enhances ferroptosis, while OE-LDLR counters these effects. These findings reveal a mechanism where PAQR3 modulates ferroptosis through LDLR/PI3K/AKT, suggesting a potential therapeutic target in DLBCL (Song et al., 2023).

## 6 Ferroptosis also regulates PI3K/Akt signaling

Current evidence supports the idea that ferroptosis can also regulate PI3K/Akt signaling as well. SLC7A11, a key component involved in redox homeostasis, is proposed to influence the activation state of AKT, a central kinase in the PI3K/AKT pathway. Inhibition of SLC7A11 appears to lead to a reduction in AKT phosphorylation, indicating a potential inhibitory effect on the PI3K/AKT pathway. While the specific molecular interactions remain unclear, the observed link underscores the crosstalk between ferroptosis, redox balance, and intracellular signaling pathways in the context of gastric cancer (Jiang Y. et al., 2023). In another study, the detailed mechanism was deciphered. It has been shown that SLC7A11 plays a crucial role in activating mTORC2/AKT pathway. Cystine, a precursor for the antioxidant glutathione, is shown to induce the activation of p38, which, in turn, regulates the assembly of mTORC2 components. Specifically, p38 mediates the phosphorylation of the mTORC2 subunit Sin1 in response to cystine stimulation. The activated mTORC2-AKT pathway, facilitated by SLC7A11 and regulated by p38-Sin1 interactions, leads to the suppression of ferroptosis (Wang G. et al., 2022). In the context of glioblastoma (GBM) development, a study highlighted the crucial role of 12-hydroxyeicosatetraenoic acid (12-HETE) in promoting GBM cell migration. ALOXE3 deficiency in GBM cells leads to a significant increase in 12-HETE secretion. Importantly, 12-HETE, acting in an autocrine manner, emerges as a key player in enhancing GBM cell migration. The mechanism involves the activation of the Gs-protein-coupled receptor (GsPCR)-PI3K-Akt pathway. This signaling cascade triggers increased expression and phosphorylation of PI3K, subsequently stimulating Akt phosphorylation at Thr308. The activation of the PI3K-Akt pathway by 12-HETE provides a molecular link between ALOXE3 deficiency, lipid metabolism, and the migratory capacity of GBM cells through PI3K-Akt pathway (Yang et al., 2021).

## 7 PI3K/Akt- mediated ferroptosis suppression in therapy resistance

**FIGURE 6:**
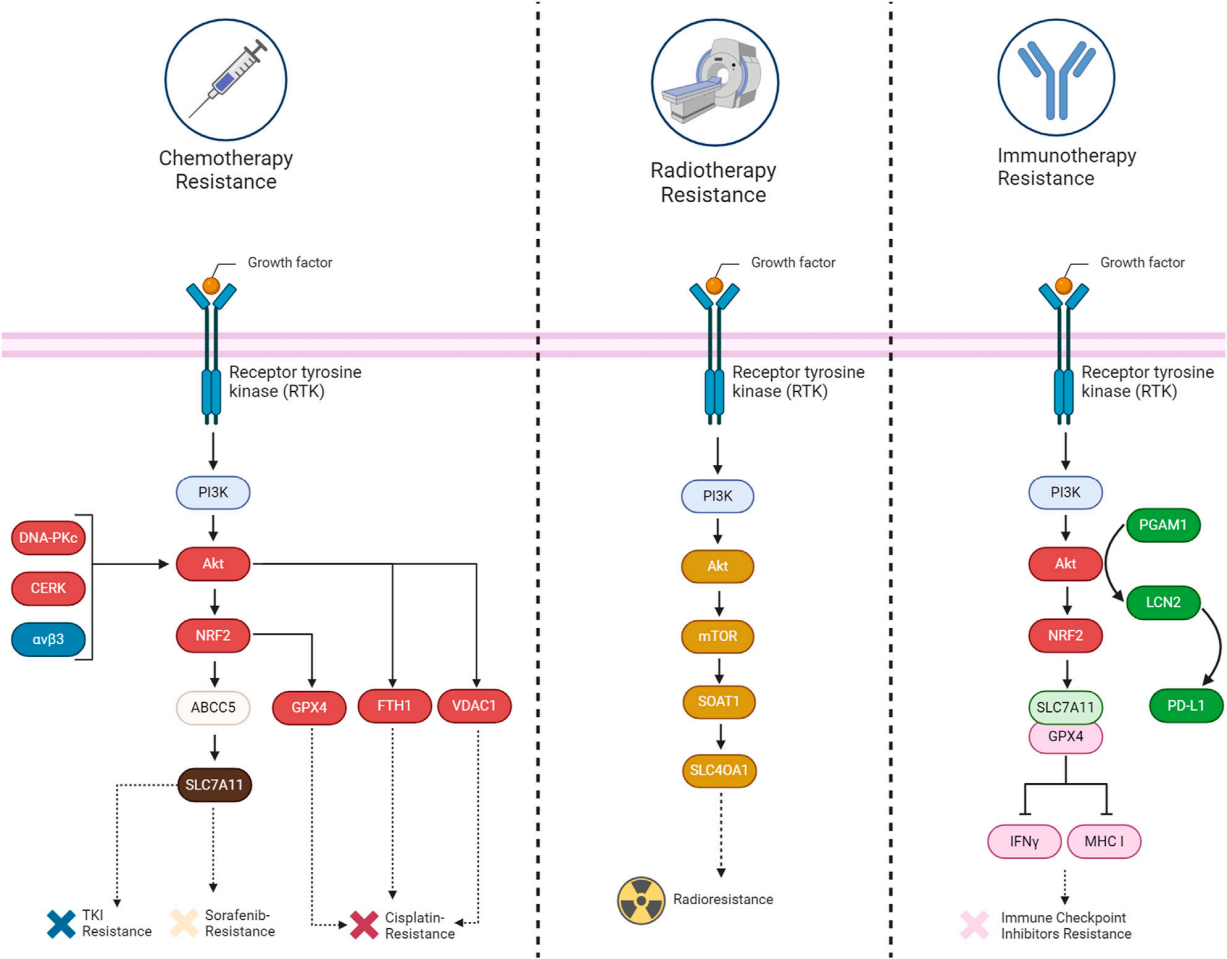
A summary of the mechanisms involved in acquiring therapy resistance (chemotherapy, immunotherapy, and radiotherapy) through the suppression of ferroptosis by the PI3K/Akt signaling pathway in cancers.

Abnormal activation of PI3K/AKT is known to cause multi-drug resistance by inhibiting apoptosis, promoting cell survival, and enhancing cancer stem cell characteristics, partially through its mediators such as ABC transporters, GSK-3β, and mTOR. Thus, inhibiting the PI3K/Akt pathway is a promising strategy to overcome therapy resistance (Liu et al., 2020). Herein, we provide evidence that shows PI3K/Akt can also cause chemotherapy, radiotherapy, and immunotherapy resistance by suppressing ferroptosis (Table 1) (Figure 6).

**TABLE 1:** PI3K/AKT regulators implicated in therapy resistance through ferroptosis suppression.

Resistance to	Main regulator	Cancer subtype	Model	Signaling axis	Highlights	References
Sorafenib	ABCC5	HCC	*in vitro* and *in vivo*	PI3K/AKT/NRF2/ABCC5/SLC7A11	After being transcriptionally activated by NRF2, ABCC5 interacts with SCL7A11 to negatively regulate ferroptosis	Huang et al. (2021)
Cisplatin	DNA-PKcs	Osteosarcoma	*in vitro* and *in vivo*	DNA-PKcs/AKT/NRF2/GPX4	GPX4 overexpression induces PD-L1 expression and chemoresistance	Zhang et al. (2023a)
Cisplatin	AKT1	Ovarian cancer	*in vitro*	AKT1/FTH1	Loss of AKT1 leads to an increase of autophagy in cisplatin-resistant ovarian cancer	Shi et al. (2023)
Cisplatin	CERK	Lung cancer with *KRAS* mutation	*in vitro* and *in vivo*	CERK/AKT/VDAC1	Inhibiting CERK reduces NSCLC cell survival and enhances cisplatin sensitivity	Vu et al. (2022)
TKI	Integrin αvβ3	HER2-Positive Breast Cancer	*in vitro* and *in vivo*	αvβ3/AKT/System Xc-	The resistance to ferroptosis is facilitated by the crosstalk between αvβ3 integrin and pathways related to iron metabolism and antioxidant response	Nagpal et al. (2023)
Radiation	SOAT1	Glioma	*in vitro* and *in vivo*	PI3K/AKT/mTOR	SOAT1 sensitizes glioma to radiation by inducing ferroptosis	Sun et al. (2023)
				/SOAT1/SLC4OA1		
Anti-PD-1 Immunotherapy	PGAM1	HCC	*in vitro* and *in vivo*	PGAM1/AKT/LCN2	PGAM1 inhibition enhances ferroptosis and promote CD8^+^ T-Cell infiltration and PD-L1 downregulation through AKT pathway	Zheng et al. (2023)
Immune Checkpoint Inhibitors (ICIs)	PI3K and HDAC	Many Cancer Cells	*in vitro* and *in vivo*	PI3K/AKT/NRF2/GPX4-SLC7A11/CD8^+^	Dual inhibition of PI3K and HDAC inhibits tumor growth via inducing ferroptosis and activation of IFNγ signaling and upregulation MHC I expression, improving ICI effects	Fan et al. (2021)

### 7.1 Chemotherapy resistance

The implication of the PI3K/Akt pathway in chemotherapy resistance is well established up to now. Several components related to this pathway have been discovered that can inhibit ferroptosis and cause chemotherapy resistance. For example, the upregulation of ABCC5 is associated with the inhibition of sorafenib-induced cytotoxicity in HCC, implying a potential role of ABCC5 in promoting cell survival pathways, such as Akt activation. Additionally, it has been revealed that ABCC5 acts as a negative regulator of ferroptosis by suppressing lipid peroxidation and increasing mitochondrial membrane potential (Huang et al., 2021). Akt signaling contributes to cisplatin resistance in ovarian cancer by inhibiting ferroptosis. DDP-resistant cells exhibit increased autophagy, and patient data indicates reduced levels of the autophagy inhibitor AKT1. Akt pathway enrichment in DDP-resistant individuals further supports its role in resistance. Restoring AKT1 decreases autophagy, indicating its involvement in the observed autophagic degradation of FTH1. This degradation leads to heightened ferroptosis sensitivity in DDP-resistant ovarian cancer cells (Shi et al., 2023). Similarly, inhibition of CERK in NSCLC cells with KRAS mutations sensitizes the cells to cisplatin through modulation of the AKT signaling pathway. The CERK inhibitor NVP-231 reduces AKT phosphorylation, leading to disruptions in the voltage-dependent anion-selective channel (VDAC)-tubulin interaction. This disruption affects mitochondrial membrane potential and increases susceptibility to ferroptotic cell death (Vu et al., 2022). In HER2-positive breast cancer cells resistant to HER2-targeting tyrosine kinase inhibitors (TKIs), an increased expression of αvβ3 integrin is observed, which is associated with sustained activation of the AKT signaling pathway. This activation of AKT by αvβ3 integrin, plays a crucial role in mediating resistance to TKIs, such as neratinib, lapatinib, and tucatinib. This integrin activation results in an upregulation of ferroportin-1, an iron exporter, leading to alterations in intracellular iron levels. The perturbation of iron homeostasis, coupled with the inhibition of System Xc^−^ activity, crucial for maintaining redox balance, contributes to the prevention of ferroptosis. The persistent activation of AKT by αvβ3 integrin further influences antioxidant responses, potentially involving the detoxifying enzyme GPX4 and the intracellular antioxidant glutathione. The integrin also appears to modulate the expression of SLC3A2/CD98, a regulatory subunit of System Xc^−^, suggesting a link to the regulation of cystine import (Nagpal et al., 2023). Likewise, the DNA-PKcs/AKT pathway is implicated in chemoresistance, and the proposed mechanism involves inhibiting ferroptosis. Persistent activation of the DNA-PKcs/AKT pathway is linked to the chemoresistance of osteosarcoma cells, and the hypothesis is that eicosapentaenoic acid (EPA) may downregulate this pathway, thereby promoting ferroptosis in response to cisplatin treatment (Zhang Y. et al., 2023).

### 7.2 Radiotherapy resistance

The involvement of the PI3K/Akt signaling pathway in tumor radioresistance is evident through diverse mechanisms. Akt, a crucial downstream mediator of PI3K, is activated, promoting cell survival and inhibiting apoptosis. This activation contributes to the resistance of cancer cells to radiation-induced cell death. Furthermore, PI3K/Akt activation plays a role in enhancing DNA repair mechanisms, facilitating cell cycle progression, and regulating autophagy. Collectively, these processes empower cancer cells to withstand the impact of radiation therapy. Researchers are actively exploring the targeting of the PI3K/Akt pathway as a potential strategy to overcome radioresistance, aiming to enhance the effectiveness of radiation treatment for cancer (Hashemi et al., 2023). Similarly, SOAT1, overexpressed in glioma tissues, is implicated in inhibiting ferroptosis and promoting radioresistance through the PI3K-AKT-mTOR signaling pathway. Positive correlations exist between SOAT1 and key components of this pathway. Silencing *SOAT1* increases sensitivity to ferroptosis, and the mTOR activator MHY-1485 reverses this effect, suggesting a connection between SOAT1 and ferroptosis via PI3K-AKT-mTOR. The mechanism through which SOAT1 influences radioresistance is not explicitly detailed, but the link between SOAT1-mediated ferroptosis modulation and the response of glioma cells to radiotherapy is clearly evident (Sun et al., 2023).

### 7.3 Immunotherapy resistance

The PI3K/Akt pathway not only contributes to cancer progression but also functions as an immunomodulator. The pathway regulates anti-tumor immunity by influencing the tumor microenvironment and immune response cell activity. Combinatorial strategies, such as combining PI3K inhibitors with immunomodulatory agents, including immune checkpoint inhibitors or small molecules targeting immunoregulatory pathways, are being explored (Caforio et al., 2021). In the context of hepatocellular carcinoma, the Akt pathway significantly modulates the response to immunotherapy by influencing ferroptosis and the tumor microenvironment. Suppression of Akt, coupled with the inhibition of PGAM1, leads to decreased lipocalin-2 (LCN2) expression, a cytokine associated with ferroptosis resistance. This dual inhibition stimulates ferroptosis in cancer cells, characterized by elevated lipid peroxidation and iron-dependent reactive oxygen species generation. Ferroptotic cancer cells release immunostimulating signals, specifically damage-associated molecular patterns (DAMPs), which are critical for inducing an inflammatory response and recruiting immune cells to the ferroptotic site. Among the identified DAMPs in the context of modified HCC cell lines, high-mobility group box 1 (HMGB1) and calreticulin (CRT) serve as signals that attract dendritic cells, macrophages, and CD8^+^ T-cells to the location of ferroptosis. These signals, associated with oxidative stress and cell death, contribute to an inflammation triggered by ferroptosis and enhance the infiltration of CD8^+^ T-cells, suggesting their potential role in antitumor immune responses (Tanaka et al., 2016; Qi and Peng, 2023; Zheng et al., 2023). Moreover, the Akt pathway regulates PD-L1 expression, a key immune checkpoint protein, and its inhibition results in reduced PD-L1 levels (Shen et al., 2023). The Interaction between the Akt pathway, ferroptosis, and immunotherapy in HCC demonstrates promising avenues for therapeutic interventions, particularly through combined targeting of these pathways to enhance treatment efficacy and patient survival (Zheng et al., 2023). BEBT-908, which is a dual PI3K/HDAC inhibitor, triggers a robust ferroptotic response in cancer cells, characterized by the reduction of SLC7A11 and GPX4, heightened lipid ROS and MDA levels, and activation of ferroptotic signaling. *In vivo* experiments demonstrate BEBT-908’s efficacy in impeding tumor growth across hematologic malignancies and solid tumors, including colorectal cancer, with a suitable pharmacokinetic profile for intravenous administration every other day. Importantly, BEBT-908 fosters a proinflammatory tumor microenvironment, facilitating the infiltration of CD8^+^ cytotoxic T cells and augmenting MHC I expression in ferroptotic cancer cells. This immunogenic property positions BEBT-908 as a promising candidate for combined therapy with immune checkpoint inhibitors (ICBs). The synergy is evident when BEBT-908 is paired with an anti-PD1 antibody, leading to sustained recoveries and the establishment of antitumor immune memory in a murine colorectal tumor model (Fan et al., 2021). A crucial association between the Akt pathway, ferroptosis, and resistance to immunotherapy has been identified in GI cancers. *ANO1* amplification, correlated with Akt pathway activation, serves as a predictor for unfavorable outcomes in immunotherapy responses. Activation of the Akt pathway and ANO1 expression is detailedly linked to the elevation of ferroptosis regulators NRF2 and SLC7A11, leading to a reduction in lipid reactive oxygen species and malondialdehyde levels. This suppression of ferroptosis contributes to the establishment of an immune-suppressive tumor microenvironment, characterized by diminished infiltration of CD8^+^ T cells. Notably, ANO1-mediated resistance to immunotherapy involves the induction of TGF-β production, recruitment of cancer-associated fibroblasts (CAFs), and the subsequent formation of an immunosuppressive milieu (Jiang F. et al., 2023; Mao et al., 2023).

## 8 Targeting PI3K/AKT to induce ferroptosis

Targeting the Akt pathway for cancer treatment is promising due to its frequent activation in different cancers and its crucial role in the PI3K/AKT/mTOR signaling pathway, which governs essential cellular functions. With its common overactivation in cancer, Akt becomes an appealing target for therapeutic intervention. Its link to drug resistance and unfavorable prognosis in specific cancers further highlights its potential as a treatment target. Various inhibitors designed to target Akt are undergoing clinical trials and exhibit promising outcomes in disrupting cancer cell growth (Song et al., 2019). Some cancer cells are more vulnerable to concurrent ferroptosis induction and PI3K/Akt inhibition. For example, the PI3K-AKT pathway is downregulated by the combined treatment with URB597 (FAAH inhibitor) and RSL3 (ferroptosis inducer) in renal cell carcinoma (RCC) cells. The downregulation of the PI3K-AKT pathway upon this treatment is suggested to contribute to the sensitivity of RCC cells to ferroptosis induced by the combination treatment (Hao et al., 2023). Similarly, several studies show that disrupting PI3K/Akt pathway activity in colorectal cancer enhances treatment efficacy with GPX4 inhibitors like RSL3 plus sorafenib (Chen H. et al., 2022; Wang L. et al., 2023). It is interesting that in glioma stem cells, ferroptosis inducers such as RSL3 cannot induce ferroptosis. Still, it can cause stem cell differentiation and reduce cell proliferation via suppressing Akt activation (Li M. et al., 2022). In contrast, several studies suggest that inhibition of the Akt pathway in malignant melanoma and triple-negative breast cancer cells sensitizes them to ferroptosis induction by RSL3 (Li P. et al., 2022; Dunsche et al., 2023; Zeng et al., 2023). This variability in responses across different cancer types underscores the complexity of the symbiosis between ferroptosis, PI3K/Akt signaling, and treatment outcomes. Herein, we show that many chemicals have been discovered that induce ferroptosis by inhibiting the PI3K/Akt pathway.

### 8.1 Synthetic PI3K/Akt axis inhibitors induce ferroptosis

In the pursuit of novel cancer therapeutics, PI3K/Akt inhibitors, crucial for disrupting cell growth and survival pathways, exhibit promise in specific cancer scenarios, especially in initial clinical phases and experimental studies. Despite encountering challenges, such as limited stand-alone effectiveness and potential toxicity concerns, ongoing investigations focus on innovative approaches, such as combining PI3K inhibitors with diverse therapeutic interventions like chemotherapy, targeted treatments, or immunotherapy (Li L. et al., 2022). This combined strategy aims to amplify treatment efficacy, expand the therapeutic scope, and minimize the risk of resistance development. The dynamic landscape of PI3K/Akt inhibitor research involves ongoing efforts to identify predictive biomarkers and refine treatment protocols tailored to different cancer types, enhancing their potential impact on cancer care (Yang et al., 2019). Therefore, it is crucial to identify novel inhibitors of this axis. In this section, we discuss the effects of these agents in the context of ferroptosis. Rimonabant, initially designed as a selective antagonist for the cannabinoid receptor type 1 (CB1), was also noted for its positive impact on the PI3K/Akt pathway. When used in combination with ferroptosis inducers like erastin or RSL3, rimonabant exhibited significant potency and a robust synergistic effect, leading to cell death in triple-negative breast cancer cell lines. The combined treatment with rimonabant and ferroptosis inducers influenced diverse cellular processes, including lipid peroxidation, depletion of GSH, and an elevation in cytosolic ROS levels (Li P. et al., 2022). The dual PI3K/HDAC inhibitor BEBT-908 orchestrates a comprehensive strategy to induce ferroptotic alterations in central nervous lymphomas. Downregulating the c-Myc oncogene disrupts cellular processes related to cysteine addiction, sensitizing cells to ferroptosis. Concurrently, BEBT-908 hampers the PI3K/AKT pathway, affecting downstream effectors crucial for protein translation. Through HDAC inhibition, it modulates histone acetylation, influencing gene expression dynamics. Noteworthy is BEBT-908’s downregulation of key ferroptosis regulators, including SLC7A11, GPX4, and Nrf2. The treatment elicits an increase in ROS production, signifying heightened oxidative stress characteristic of ferroptosis. Furthermore, BEBT-908 induces alterations in iron metabolism-related genes, exemplified by the upregulation of transferrin receptor (TRFC). These combined actions create a cellular milieu conducive to ferroptosis, marked by lipid peroxidation and culminating in cell death (Fan et al., 2021; Wang N. et al., 2023).

The combination treatment with FAAH inhibitors, exemplified by URB597, and ferroptosis inducers has been shown to effectively inhibit the PI3K/Akt pathway in the context of renal cell carcinoma. Through a multifaceted approach, this combination downregulates the PI3K/Akt pathway, as evidenced by KEGG analysis and validated by qRT-PCR, thereby influencing global gene expression related to cell proliferation, cell cycle, and cell migration. The synergy of FAAH inhibition and ferroptosis induction results in enhanced inhibition of RCC cell growth, colony formation, and migration. Experiments with specific inhibitors (PD98059 for ERK1/2 and LY294002 for Akt) support the involvement of the PI3K/Akt pathway in FAAH-modulated sensitivity to ferroptosis. This combination treatment, which elaborately regulates key pathways associated with cancer progression, presents a promising therapeutic strategy for inhibiting RCC tumor growth and metastasis. Dihydroartemisinin (DHA), a derivative of artemisinin from the sweet wormwood plant, demonstrates antimalarial efficacy and shows promise in treating certain cancers, particularly those resistant to conventional therapies. Emerging research indicates that DHA induces ferroptosis. DHA contributes to ferroptosis by elevating ROS levels, depleting glutathione, disrupting iron homeostasis, and causing mitochondrial changes. Signaling pathways, such as AKT/mTOR, may be modulated by DHA, influencing cellular responses to oxidative stress. The potential of DHA to induce ferroptosis highlights its relevance as a novel therapeutic strategy, particularly in combatting drug-resistant cancers (Zhang X. et al., 2022). Notably, Fe3O4-PGA nanoparticles can be utilized to encapsulate DHA through a solvothermal synthesis approach. These nanoparticles possess a consistent structure and exhibit superparamagnetic characteristics, allowing controlled DHA release within the tumor microenvironment (Li et al., 2024). DHA, upon release, instigates ferroptosis via a Fenton-like reaction, producing ROS that initiates lipid peroxidation. The liberated DHA markedly diminishes the levels of phosphorylated AKT (p-AKT) and phosphorylated mTOR (p-mTOR), suggesting the involvement of the PI3K/Akt/mTOR/GPX4 pathway in ferroptosis induction. These nanoparticles demonstrate heightened cytotoxicity against triple-negative breast cancer cells compared to free DHA, with ferroptosis and apoptosis identified as the primary cell death mechanisms. Comprehensive lipidomics and transcriptomics analyses corroborate the engagement of ferroptosis-related pathways, highlighting the potential utility of Fe3O4-PGA-DHA nanoparticles as a therapeutic approach for TNBC, particularly when combined with Fe3O4-PASP-DOX to augment cytotoxic effects (Zhang J. et al., 2023).

Fatostatin exerts its influence on glioblastoma multiforme cells through a complex mechanism. It dose-dependently inhibits the Akt signaling pathway, resulting in reduced phosphorylation of Akt, mTORC1, and 4EBP1. This inhibition disrupts the Akt/mTORC1/4EBP1 axis, crucial for protein synthesis. Consequently, fatostatin adversely affects the protein levels of GPX4, a critical regulator of ferroptosis. Simultaneously, fatostatin prompts ferroptosis in GBM cells, as evidenced by heightened lipid peroxidation and distinctive morphological alterations. The attenuation of Akt/mTORC1/4EBP1 signaling plays a role in the observed reduction in GPX4 and the initiation of ferroptosis in GBM cells (Cai et al., 2023).

Lorlatinib, an FDA-approved inhibitor primarily targeting ALK/ROS1 tyrosine kinases, is under investigation for its impact on stearoyl-CoA desaturase (SCD) expression in melanoma cells. It has been revealed that lorlatinib’s inhibition of PI3K/AKT/mTOR pathways contributes to reducing SREBP1 activity and suppressing fatty acid metabolism. This cascade leads to diminished SCD expression, a crucial enzyme in lipid metabolism. Lorlatinib’s influence on SCD is associated with its ability to sensitize melanoma cells to ferroptosis. The kinase inhibition spectrum of lorlatinib extends to IGF1R (insulin-like growth factor 1 receptor), identified as a major contributor to lorlatinib-induced sensitivity to ferroptosis. Combining lorlatinib with GPX4 knockout, a regulator of ferroptosis, significantly suppresses melanoma growth *in vivo*. These findings underscore lorlatinib’s potential therapeutic impact on lipid metabolism modulation and ferroptosis induction in melanoma (Zeng et al., 2023).

Lastly, some evidence suggests that anesthetics can also possess anti-cancer properties by inducing ferroptosis (Abudurousuli et al., 2023). This becomes more intriguing when inhibiting PI3K/Akt by local anesthetic agents, such as bupivacaine and ropivacaine, can trigger these ferroptotic changes (Hao et al., 2022; Lu et al., 2022). This signaling pathway appears to function as a central regulatory pathway for controlling ferroptosis in cancer cells.

### 8.2 Natural products targeting PI3K/AKT/ferroptosis axis

Natural products have been actively explored as potential modulators of the PI3K/Akt pathway. Compounds such as curcumin from turmeric, resveratrol from red grapes, quercetin from fruits and vegetables, EGCG from green tea, genistein from soy, and berberine from various plants have shown promise in preclinical studies for their ability to inhibit the PI3K/Akt pathway, leading to anti-cancer effects. These natural products represent a diverse array of compounds with potential therapeutic benefits (Bai et al., 2022; Gao et al., 2022; Tabnak et al., 2022; Tewari et al., 2022). Extensive naturally derived compounds that induce ferroptosis by inhibiting the PI3K/Akt pathway in cancers have been discovered. These compounds, along with their targets, are summarized in Table 2. As seen, many of the naturally derived compounds can inhibit tumor growth *in vivo* and *in vitro*, synergizing with conventional therapeutic drugs such as cisplatin (osteosarcoma and gastric cancer), gefitinib (in lung cancer), and docetaxel (prostate cancer). Targeting the PI3K/Akt/Ferroptosis axis with these compounds is a promising strategy against gastrointestinal cancers such as colon and gastric cancers.

**TABLE 2:** Natural products targeting PI3K/Akt and ferroptosis simultaneously.

Name	Source	Cancer	Pathway	Effects	Model	References
Zerumbone	Zingiber zerumbet Smith	Lung Cancer	AKT/STAT3/SLC7A11	In combination with Gefitinib inhibits proliferation and tumor growth	*In vivo* and *In vitro*	Wang et al. (2023a)
Banxia Xiexin Decoction	Multiple sources	Colon Cancer	PI3K/AKT/mTOR/ULK1,2/NCOA4/FTH1	Inhibits proliferation and migration	*In vivo* and *In vitro*	Wang et al. (2024a)
				Increases iron and ROS levels		
				Induces ferritinophagy		
Yi-qi-hua-yu-jie-du decoction	Multiple sources	Gastric Cancer (DDP-Resistant)	AKT/GSK3β/NRF2/GPX4	Enhances cisplatin efficacy	*In vivo* and *In vitro*	Huang et al. (2024)
				Inhibits proliferation		
				No damage to the liver or kidney *in vivo*		
Red ginseng polysaccharide	Red ginseng	Gastric Cancer	AQP3/PI3K/AKT/AKT/SLC7A11	Inhibits proliferation	*In vivo* and *In vitro*	Wang et al. (2024b)
				Reduce tumor weight and volume		
Eicosapentaenoic acid	Fish oil	Osteosarcoma	DNA-PKcs/AKT/NRF2	Enhances cisplatin efficacy	*In vivo* and *In vitro*	Zhang et al. (2023a)
				Reduce tumor weight and volume		
Osthole	Cnidium spp. and other Apiaceous plants	Colon Cancer	AMPK/Akt/mTOR/NCOA4	Inhibits malignant behaviors	*In vivo* and *In vitro*	Zhou et al. (2023)
				Increases the anti-cancer effects of cetuximab in KRAS mutant cells		
Docosahexaenoic acid	Fish oil	Prostate Cancer	PI3K/AKT/Nrf2/GPX4	Combination of DCA with docetaxel is more effective	*In vitro*	Shao et al. (2022)
				In suppressing proliferation and colony formation		
Curcumin	Curcuma longa	Colon Cancer	PI3K/Akt/mTOR/GPX4	Inhibits proliferation	*In vitro*	Chen et al. (2023c)
6-shogaol	Ginger	Endometrial carcinoma	PI3K/AKT/GPX4	Inhibits invasion and migration	*in vitro* and *in vivo*	Ma et al. (2022)
Nitidine chloride	Zanthoxylum nitidum	Multiple myeloma	ABCB6/PI3K/AKT/NRF2/GPX4	Reduced the volume and weight of tumors	*in vitro* and *in vivo*	Yin et al. (2023)

## 9 Conclusion

This review explored the interaction between the PI3K/Akt pathway and ferroptosis. The activation of PI3K/Akt has a substantial inhibitory effect on ferroptosis in numerous cancer cells. The components of this signaling pathway directly impact the expression of ferroptosis regulators like GPX4, SLC7A11, NRF2, ferritin, and ACSL4, thus influencing various aspects of ferroptosis, including amino acid, iron, and lipid metabolism. The involvement of downstream targets of PI3K/Akt, such as GSK3β, mTORC1, STAT3, and HIF1α, in ferroptosis regulation is well-documented in the literature. Additionally, ferroptosis can reciprocally influence the PI3K/Akt pathway, with protective molecules against ferroptosis, such as SLC7A11, being capable of activating it. The PI3K/Akt/Ferroptosis axis holds significant importance in cancer resistance, impacting chemotherapy, radiotherapy, and immunotherapy. This axis, characterized by the interplay between PI3K/Akt signaling and the regulation of ferroptosis, contributes to resistance mechanisms in various ways. In chemotherapy, Akt activation inhibits ferroptosis, promoting cell survival and resistance to cytotoxic treatments. The PI3K/Akt pathway also plays a role in radioresistance by influencing cell survival, DNA repair, and autophagy. In immunotherapy, the axis acts as an immunomodulator, affecting the tumor microenvironment and anti-tumor immunity. Inhibition of Akt stimulates ferroptosis, enhancing the tumor microenvironment with immune cell infiltration and reducing immune checkpoint protein PD-L1 levels. Advancements in inhibiting the PI3K/Akt pathway and inducing ferroptosis for cancer treatment are promising and multifaceted. Synthetic inhibitors targeting PI3K/Akt, undergoing clinical trials, aim to disrupt aberrant Akt pathway activation in diverse cancers. Combination therapies, integrating PI3K/Akt inhibitors with chemotherapy, targeted treatments, or immunotherapy, are actively explored to enhance treatment efficacy and mitigate resistance risks. It should be noted that ferroptosis inducers, such as RSL3 and Erastin, are still being explored in preclinical and cell line studies. No clinical evidence currently exists regarding their combined effects with PI3K/AKT inhibitors in cancers. Therefore, it is recommended to examine the efficacy of these agents in clinical settings. Experimental drugs like BEBT-908 and rimonabant, along with natural products such as curcumin and osthole, exhibit the potential to induce ferroptosis by targeting the PI3K/Akt pathway. In addition, nanoparticle-based strategies, like Fe3O4-PGA encapsulating ferroptosis-inducing agents, offer targeted drug delivery. This evolving landscape highlights the dynamic progress in understanding the intricate interplay between PI3K/Akt inhibition and ferroptosis induction, holding substantial potential for future clinical applications in cancer therapy.
